# Modulation of Pulmonary Toxicity in Metabolic Syndrome Due to Variations in Iron Oxide Nanoparticle-Biocorona Composition

**DOI:** 10.3390/nano12122022

**Published:** 2022-06-11

**Authors:** Li Xia, Saeed Alqahtani, Christina R. Ferreira, Uma K. Aryal, Katelyn Biggs, Jonathan H. Shannahan

**Affiliations:** 1 School of Health Sciences, College of Health and Human Sciences, Purdue University, West Lafayette, IN 47907, USA; xia104@purdue.edu (L.X.); salqaht@purdue.edu (S.A.); biggs23@purdue.edu (K.B.); 2 Life Science and Environment Research Institute, King Abdulaziz City for Science and Technology (KACST), Riyadh 12354, Saudi Arabia; 3 Purdue Metabolite Profiling Facility, Purdue University, West Lafayette, IN 47907, USA; cferrei@purdue.edu; 4 Purdue Proteomics Facility, Bindley Bioscience Center, Purdue University, West Lafayette, IN 47907, USA; uaryal@purdue.edu; 5 Department of Comparative Pathobiology, Purdue University, West Lafayette, IN 47907, USA

**Keywords:** iron oxide nanoparticles, inhalation, metabolic syndrome, bronchoalveolar lavage fluid, pulmonary, inflammation, mechanisms, susceptibility

## Abstract

Nanoparticles (NPs) interact with biomolecules by forming a biocorona (BC) on their surface after introduction into the body and alter cell interactions and toxicity. Metabolic syndrome (MetS) is a prevalent condition and enhances susceptibility to inhaled exposures. We hypothesize that distinct NP-biomolecule interactions occur in the lungs due to MetS resulting in the formation of unique NP-BCs contributing to enhanced toxicity. Bronchoalveolar lavage fluid (BALF) was collected from healthy and MetS mouse models and used to evaluate variations in the BC formation on 20 nm iron oxide (Fe_3_O_4_) NPs. Fe_3_O_4_ NPs without or with BCs were characterized for hydrodynamic size and zeta potential. Unique and differentially associated proteins and lipids with the Fe_3_O_4_ NPs were identified through proteomic and lipidomic analyses to evaluate BC alterations based on disease state. A mouse macrophage cell line was utilized to examine alterations in cell interactions and toxicity due to BCs. Exposures to 6.25, 12.5, 25, and 50 μg/mL of Fe_3_O_4_ NPs with BCs for 1 h or 24 h did not demonstrate overt cytotoxicity. Macrophages increasingly associated Fe_3_O_4_ NPs following addition of the MetS BC compared to the healthy BC. Macrophages exposed to Fe_3_O_4_ NPs with a MetS-BC for 1 h or 24 h at a concentration of 25 μg/mL demonstrated enhanced gene expression of inflammatory markers: *CCL2*, *IL-6*, and *TNF*-α compared to Fe_3_O_4_ NPs with a healthy BC. Western blot analysis revealed activation of STAT3, NF-κB, and ERK pathways due to the MetS-BC. Specifically, the Jak/Stat pathway was the most upregulated inflammatory pathway following exposure to NPs with a MetS BC. Overall, our study suggests the formation of distinct BCs due to NP exposure in MetS, which may contribute to exacerbated inflammatory effects and susceptibility.

## 1. Introduction

Nanoparticles (NPs) can be synthesized with a variety of novel and modifiable physicochemical properties and their application is benefiting numerous fields such as medicine, agriculture, manufacturing, and consumer products. Safety assessments have demonstrated that inhaled NPs efficiently deposit within the respiratory tract causing a variety of adverse health effects including inflammation, apoptosis, oxidative stress, damage to the epithelial air interface, fibrotic changes, impaired pulmonary function, lung injury, and systemic effects [1,2]. Iron oxide (Fe_3_O_4_) NPs are highly utilized with broad applications due to their superparamagnetic characteristics and are considered comparably more biocompatible than many other NPs. This results in their utilization and proposed usage in biomedicine (drug delivery systems, gene carriers, magnetic probes, vaccine adjuvants, and others), as well as several industrial processes (colorants, chemical reaction catalysts, and fuel production) [3,4,5,6,7]. With these diverse applications, their production has continued to increase in recent years.

In industrial settings, workers are at risk for Fe_3_O_4_ NPs exposure during their production and use. Specifically, an assessment of occupational exposure to Fe_3_O_4_ NPs in a production facility demonstrated mass concentrations of 335 μg/m^3^ in the personal breathing samples of workers without local engineering controls [8]. Another assessment determined respirable mass concentrations between 1 μg/m^3^ and 1.07 μg/m^3^ [9]. These exposure assessments determined Fe_3_O_4_ NP exposures were variable based on worker duties, however, were below the exposure limits established by regulatory agencies (Occupational Safety and Health Agency—10 mg/m^3^ averaged over an 8-h workshift; National Institute of Occupational Safety and Health—5 mg/m^3^ averaged over a 10-h workshift; American Conference of Government Industrial Hygienists—5 mg/m^3^ averaged over an 8-h workshift [10,11]. Evaluation of oxidative stress biomarkers in exhaled breath condensate demonstrated significant increases in workers manufacturing Fe_3_O_4_ NPs in a pigment factory [12]. Additionally, in vivo toxicity evaluations using rodent models have observed inflammation, oxidative stress, and pulmonary injury following iron oxide NP inhalation exposures [13,14].

Most toxicity assessments have examined NP physicochemical properties to identify potential adverse health outcomes, however, these results may not be applicable to large portions of our population. Specifically, in the United States, 6 in 10 adults suffer from a chronic disease, while 4 in 10 have 2 or more [15]. Pre-existing diseases enhance sensitivity to inhalation exposure-induced health effects. Metabolic syndrome (MetS) is a significant public health concern affecting 34.2% of U.S. adults, 50% of individuals over 60 years old, and 9.8% of children [16]. MetS is an aggregate of clinical conditions including abdominal obesity, systemic hypertension, insulin resistance, hypertriglyceridemia, and low high-density lipoprotein (HDL) predisposing individuals to cardiovascular disease, type 2 diabetes mellitus, cancer, and other serious chronic diseases [17]. Human studies have established individuals suffering from MetS are increasing susceptible to inhalation exposures. For example, individuals with MetS exposed to World Trade Center dust exhibited diminished lung function compared to healthy individuals [18]. Our recent research has demonstrated enhanced susceptibility to inflammation in a MetS mouse model compared to a healthy following pulmonary exposure to silver nanoparticles [19,20]. Although, susceptibility is recognized, the mechanisms are unknown by which underlying disease states such as MetS enhance toxicity following inhalation exposures.

When NPs are introduced into the body, they rapidly become coated with biomolecules forming a NP-biocorona (BC). This BC alters NP characteristics and subsequent cell interactions, influencing NP functionality, biodistribution, clearance, and toxicity. Formation of the BC is governed by NP properties, time, and the biological environment. Numerous diseases alter the biological milieu which may influence BC formation and contribute to susceptibility differences. Our previous research demonstrated unique BCs form in obese human serum compared to healthy resulting in altered cellular interactions and immune responses [19,20,21,22]. MetS modifies the biomolecule content within the circulation and also extend to changes in the lung. These alterations in protein and lipid content may influence susceptibility to NP-induced pulmonary toxicity through the formation of distinct BCs [23,24,25,26].

We hypothesize that distinct BCs form on the surface of NPs following their introduction into the lung due to underlying disease states such as MetS resulting in enhanced toxicity. To examine the contribution of the BC as a mechanism facilitating susceptibility, bronchoalveolar lavage fluid was isolated from healthy and MetS mice and used to generate NP-BCs ex vivo. An integrative proteomic and lipidomic approach was utilized to characterize differential biomolecule content of the BCs that formed due to healthy and MetS conditions. Alterations in macrophage interactions and inflammatory responses were evaluated in vitro to determine the contribution of variations in the BC. Overall, this study evaluates a specific mechanism which may contribute to enhanced susceptibility to particulate exposures occurring in prevalent underlying diseases such as MetS.

## 2. Materials and Methods

### 2.1. Animal Model

C57BL/6J mice (Jackson Labs, Bar Harbor, ME, USA) at 6 weeks of age were placed on either a healthy diet with 10% of kcal coming from fat (D12450B, Research Diets Inc., New Brunswick, NJ, USA), containing 51.6 mg/kg cholesterol or a high-fat western diet with 60% of kcal coming from fat (D12450B, Research Diets Inc.), containing 279.6 mg/kg cholesterol. This is a well-established model for producing mice with metabolic syndrome (MetS) [27,28]. Our previous work has generated consistent results with very little variability in inducing MetS by using these diets [19,20,21]. Specifically, we have established these diets result in approximately a 50% weight gain and over a 30% increase in total cholesterol levels and 6% in insulin without altering hemoglobin A1C [19,21]. Mice were housed in a room with constant temperature, 12/12-h light/dark cycle. All animal procedures were conducted in accordance with the National Institutes of Health guidelines and approved by the Purdue University Animal Care and Use Committee.

### 2.2. Necropsy and BALF Collection

Mice were necropsied at 18 weeks of age to collect bronchoalveolar lavage fluid (BALF) from the whole lung. Briefly, the lung was lavaged once with cold phosphate buffered saline (PBS) at a volume of 35 mL/kg of body weight. All individual BALF samples from the groups were pooled together and centrifuged at 1000× *g* at 4 °C for 10 min. The biomolecule-rich supernatant was isolated, aliquoted into individual volumes of 200 μL and stored at −80 °C for utilization in forming BCs.

### 2.3. Model Characterization

Blood was collected from healthy and MetS mice via cardiac puncture. Blood samples were centrifuged at 3500× *g* at 4 °C for 10 min and serum was isolated. Collected serum samples were used to evaluate total cholesterol, high-density lipoprotein (HDL), and low-density (LDL)/very-low-density lipoprotein (VLDL) levels to verify groups. All serum endpoints were quantified using commercially available kits (Bioassay Systems, Hayward, CA, USA) via manufacturer’s protocols.

### 2.4. NP-BC Formation and Characterization

For this evaluation, 20 mg/mL iron oxide nanoparticles (Fe_3_O_4_ NPs) with a hydrodynamic diameter of 20 nm in aqueous 2 mM sodium citrate (NanoComposix, San Diego, CA, USA) were utilized. To formulate the NP-BCs, 125 μL of Fe_3_O_4_ NPs at a concentration of 1 mg/mL were diluted with 125 μL deionized water (di H_2_O), centrifuged at 15,000 rpm for 30 min, and the supernatant removed. The isolated Fe_3_O_4_ NPs were then resuspended thoroughly with either 500 μL BALF from healthy or MetS mice. NPs were then incubated at 4 °C for 8 h while rotating. Following the incubation, NPs were washed 3 times via centrifugation at a speed of 15,000 rpm and resuspended in water at their original concentration of 1 mg/mL. Original NPs and those with the BCs were diluted to a concentration of 25 μg/mL and characterized via assessment of hydrodynamic size, polydispersity index, and ζ-potential (ZetaSizer Nano, Malvern Panalytical Ltd., Malvern, UK) (n = 3/particle). These methods have been previously utilized by our laboratory and other to characterize and verify NPs and NP-BCs [19,20,21,22,29,30].

### 2.5. BC Characterization-Proteomics

Protein components from healthy and Mets BCs were isolated and analyzed by liquid chromatography-tandem mass spectrometry (LC-MS/MS) using similar method as described in our previous studies [22,31,32]. Briefly, following the isolation of NPs with BCs by centrifugation at 21,130× *g*, samples were resuspended in 8M urea for 45 min to disassociate proteins from NPs by centrifugation at 15,000× *g*. The supernatant containing proteins isolated were reduced by incubating in 10 mM dithiothreitol (DTT) for 1 h at 37 °C followed by alkylation by incubating in 30 mM iodoacetamide (IAA) for 45 min in dark at room temperature. Then samples were digested into peptides overnight at 37 °C incubating with trypsin at 1:25 enzyme:substrate ratio. Digested peptides were purified (desalted) by using UltraMicroSpin columns (The Nest Group, Ipswich, MA, USA) following the protocol, provided by the manufacturer. Purified peptides were dried under vacuum, resuspended in 0.1% formic acid (FA) and 3% acetonitrile to a final concentration of 0.2 μg/μL and 5 μL (1 μg) was used for LC-MS/MS analysis using a Dionex UltiMate 3000 RSLC Nano LC System attached to the Q Exactive™ HF Hybrid Quadrupole-Orbitrap Mass Spectrometer (Thermo Scientific, Waltham, MA, USA). Peptides were separated using trap and analytical columns using the gradient method as described in the previous publication [31]. Specifically, peptides were first loaded into the trap cartridge at 5 μL/min flow rate using a loading pump and washed for 5 min using 2% acetonitrile in HPLC-grade water and separated in an analytical column using a 120-min gradient method. Buffer A was a 2% acetonitrile and 0.1% FA, and buffer B was 80% acetonitrile and 0.1% FA. Peptides were separated as follows: a linear gradient of 5% to 30% of buffer B in 80 min, 45% of B in 91 min, and 100% of B in 93 min. Then, buffer B was held at 100% of B for 7 min until switching back to 5% of B and held at 5% of B for next 20 min. After each run, columns were cleaned with a linear 30-min gradient of 5% to 100% of B 2 times to remove any residual peptides, and then equilibrated for another 30-min before loading the next sample to reduced sample carryover. The QE-HF mass spectrometer was operated in Data Dependent Acquisition (DDA) mode with MS1 resolution of 120,000 and MS2 resolution of 15,000 at 200 *m*/*z*. The MS instrument was calibrated at the start of the experiment and then in every 72 h or as needed. The reproducibility of the mass spectrometer was evaluated and monitored by running Hela digest as standards.

All mass spectrometer data were processed by the MaxQuant computational proteomics platform version 1.6.3.3 (Computational Systems Biochemistry, Martinsried, Germany) via the iBAQ (intensity based absolute quantitation) method. iBAQ intensities of proteins detected in three of four replicates from each group and with at least 2 MS/MS counts were considered as present for comparisons. Data were searched at 1% FDR (false discovery rate) for both peptide spectral match and proteins. Common contaminant proteins were removed before any subsequent analyses. A Venn diagram was produced using Venny 2.1.0 (http://bioinfogp.cnb.csic.es/tools/venny, accessed on October 2021) to identify similarities and differences between the BCs. To quantify differences in abundance of shared proteins identified in both the health and MetS BCs, fold changes of intensities were calculated comparing each replicate to the mean intensity of the healthy group. One-way ANOVAs with a Tukey’s posthoc test were utilized to determine significantly different proteins using a *p*-value < 0.05.

### 2.6. BC Characterization-Lipid Profiling

To assess the lipid components of the NP-BCs a separate set of samples were produced using the same incubation conditions. The multiple reaction monitoring (MRM) profiling method was used to profile ceramides (Cer), phosphatidylcholines and sphingomyelins (PC-SM), phosphatidylethanolamines (PE), phosphatidylglycerols (PG), phosphatidylinositols (PI), phosphatidylserines (PS), cholesteryl esters (CE), triacylglycerols (triglycerides) (TAG), and acyl-carnitines (AC) and present by their relative amounts [33]. Previously we have performed this procedure to examine components of the NP-BC following incubation in serum [29]. Briefly, 125 μg Fe_3_O_4_ NPs were incubated in 500 μL BALF. Following BC formation and isolation of NPs by centrifugation, NP-associated lipids were extracted from the NP surface via a modified Bligh-Dyer lipid extraction method [34]. The MRM-profiling methods and instrumentation used have been recently described in previous reports [33,35,36,37,38]. Specifically, isolated NP-BCs (described previously) were resuspended in 200 μL water following by adding 125 μL of chloroform and 250 μL of methanol. The mixture was thoroughly. Then 125 μL of water and 125 μL of chloroform were added to it. Centrifugation were unitized at 16,000× *g* for 10 min to separate the solution to two phases. The cloudy bottom phase was the organic chloroform phase containing the lipids, was isolated and transferred to a clean microtube. The extracts were dried using a speed vacuum centrifuge. Dried samples were resuspended in 50 µL of methanol/chloroform 3:1 (*v*/*v*) and a 250 µL of 3:6.65:0.35 (*v*/*v*/*v*) mixture of acetonitrile, methanol, and ammonium acetate 300mM to obtain a stock solution. The stock solution was further diluted 200 times for analysis. Then, 8 μL of each sample was injected using a micro-autosampler (G1277A) directly to the ESI source of an Agilent 6410 triple quadrupole mass spectrometer (Agilent Technologies, Santa Clara, CA, USA). A capillary pump was connected to the autosampler and operated at a flow rate of 7 μL/min and pressure of a 100 bar. Capillary voltage on the instrument was 5 kV and the gas flow 5.1 L/min at 300 °C. The MS data obtained from these methods was processed using an in-house script to obtain a list of MRM transitions with their respective sum of absolute ion intensities over the acquisition time. The parent *m*/*z* of the MRMs screened were based on the Lipid Maps Structure Database (https://www.lipidmaps.org/ accessed on August 2021), and the product ions were related to class-diagnostic fragments [39]. Free fatty acids were monitored only by the parent mass. The respective ion intensity values were then considered for data analysis. There were 4 samples in each BC group. MRM signals were required to be 30% above the blank in all replicates to be considered for data analysis. For univariate analysis, *p*-value < 0.05 was considered significant. Statistical analysis was performed utilizing Metaboanalyst 5.0 (http://www.metaboanalyst.ca/ accessed on August 2021). Data on the relative amounts were auto scaled to obtain a normal distribution, and evaluated by univariate analysis, principal component analysis (PCA), and cluster analysis/heatmap. Informative lipids were analyzed according to class, fatty acyl residue chain length and unsaturation level. Lipids which were significantly different with big fold change between two BCs were also graphed and shown through a histogram.

### 2.7. Cell Culture and Exposure

A representative mouse macrophage cell line (RAW 264.7) (ATCC, Manassas, VA, USA) was utilized in this study to examine BC-induced differences in cellular interactions and toxicity. Cells were cultured and maintained in culture petri dishes using Dulbecco’s modified Eagle’s medium supplemented with 10% fetal bovine serum and 1% antibiotic at 37 °C and 5% CO_2_. Cells were seeded in either 6-, 24-, or 96-well plates as needed for experiments. When cells reached 90% confluency, they were exposed to NPs with or without BCs at concentrations of 0, 6.25, 12.5, 25, or 50 μg/mL for 1 h or 24 h in serum-free media (SFM). SFM was used to maintain the preformed BC on the surface of NPs, however, limited our assessments to acute time points.

### 2.8. Assessment of NP-BC Cytotoxicity

RAW 264.7 cells were exposed to Fe_3_O_4_ NPs with BCs at concentrations of 0, 6.25, 12.5, 25, or 50 μg/mL for 1 h or 24 h, and cytotoxicity was assessed through use of the Thiazolyl Blue Tetrazolium Bromide (MTT) cell proliferation assay (Sigma Aldrich, St. Louis, MO, USA). Briefly, after exposure, cells were incubated with the MTT reagent at a concentration of 0.5 mg/mL for 3 h in the 37 °C incubator. DMSO was added to dissolve insoluble purple crystals that had form during the MTT incubation. The associated color change was measured by a plate reader (Molecular Devices, San Jose, CA, USA) at 570 nm. Comparison to a control group allowed for determination of changes in viability due to exposure (n = 4/group). Three technical replicates were present on each plate, and averaged together to create an individual sample. Untreated cells in SFM were used as controls for the MTT assay.

### 2.9. Macrophage Association of NPs

Macrophages were seeded in 6-well plates, exposed to NP-BCs at a concentration of 25 μg/mL for 24 h, and NP-cellular associations were evaluated. After NP-BC exposure, cells were rinsed twice by cold PBS, and then lysed by adding cold radio-immunoprecipitation assay (RIPA) buffer. Collected cell lysates were centrifuged at 350× *g* for 5 min, and the supernatant was used for total protein concentration measurement by Pierce BCA Protein Assay Kit (Thermo Fisher Scientific, Rockford, IL, USA). After total protein concentration was measured, the sample was vortexed well, and metal content was measured by an X-ray fluorescence (XRF) analyzer (Thermo Scientific, Billerica, MA, USA). This analyzer measures the fluorescent X-rays emitted from a sample when excited by a primary X-ray source. Each of the elements contained in a sample produce uniquely characteristic fluorescent X-rays. The XRF has the capacity to measure multiple metals simultaneously metals (Fe, Ag, Cd, Te, Sn, Sb, Ca, K, Ba, Sc, Ti, V, Cr, Mn, Co, Cu, Zn, Hg, As, Se, Pb, Sr, Mo, Rb, and others), allowing for the quantitative determination of NP-cellular association (n = 5/group). Untreated cells were used as controls for XRF analyzer measurements. To qualitatively examine NP-cell associations, Cytoviva enhanced hyperspectral dark field microscopy was utilized [20,40]. Briefly, following exposure to SFM (controls) or 25 μg/mL NPs with BCs for 24 h, macrophages were stained with DAPI to identify the nucleus through fluorescent imaging. Then the same macrophages were imaged using dark field microscopy to identify NPs. Both images were captured at a magnification of 100× and then overlayed to qualitatively confirm differential Fe_3_O_4_ NPs association due to addition of BCs.

### 2.10. Examination of Inflammation

This assessment aimed to examine BC-induced variations in inflammation following exposure. Macrophages were exposed to 25 μg/mL Fe_3_O_4_ NPs with BCs for 1 h or 24 h. Cells were harvested in Trizol (Invitrogen, Carlsbad, CA, USA), and total RNA was isolated by Direct-zolTM RNA MiniPrep Kits (Zymo Research, Irvine, CA, USA) following the manufacturer’s instructions. After RNA extraction, the concentration was measured by Nanodrop (Thermo Scientific, Hercules, CA, USA). A total amount of 1000 ng of RNA was mixed with 5× iScript reaction mix, iScript reverse transcriptase and nuclease-free water to reach a total volume of 20 μL. RNA was reversely transcribed into cDNA by using the cDNA synthesis kit (Bio-Rad, Hercules, CA, USA) in a thermal cycler (Eppendorf, Enfield, CT, USA). After cDNA synthesis, alterations of inflammatory gene expression and oxidative stress markers including *Tumor necrosis factor alpha* (*TNF*-α), *interleukin-6* (*IL-6*), *C-X-C Motif Chemokine Ligand 2* (*CXCL-2*), *vascular cell adhesion molecule 1* (*VCAM1*), *C-C Motif Chemokine Ligand 2* (*CCL2*), and *Heme Oxygenase 1* (*HMOX 1*) were assessed utilizing mouse-specific primers (Qiagen, Hilden, Germany) through quantitative real-time reverse transcriptase polymerase chain reaction (real-time RT PCR). *Glyceraldehyde 3-phosphate dehydrogenase* (*GAPDH*) was used as the internal control for all gene expressions. Relative fold gene expression of samples among different groups were calculated by using the delta-delta Ct method (2^−∆∆Ct^) (n = 5/group). Untreated cells in SFM were used as controls. Additionally, 2 technical replicates for each sample were used when running real-time RT PCR.

### 2.11. BC-Induced Regulation of Inflammatory Signaling

Expression and activity of key components of inflammatory signaling pathways including nuclear factor (NF)-κB, p38/mitogen-activated protein kinase (MAPK), Janus kinase (JAK)/signal transducer activator of transcription 3 (STAT3) and ras/raf/mitogen-activated protein kinase/ERK kinase (MEK)/extracellular-signal-regulated kinase (ERK) were examined. RAW 264.7 cells were seeded in 6-well plates and exposed to 25 μg/mL NPs with BCs for 24 h. Cells were harvested and lysed in RIPA buffer containing protease inhibitor (Thermo Scientific, Hercules, CA, USA). Total protein concentrations were quantified using a Pierce BCA Protein Assay Kit (Thermo Scientific, Rockford, IL, USA). Then, 20 µg of protein was mixed with an equal volume of 2× sample buffer, loaded on a 10% SDS-PAGE gel, underwent electrophoresis, and transferred to a polyvinylidene difluoride (PVDF) membrane at a consistent current of 0.3A for 1.5 h. Following protein electrotransfer, blot was incubated in Ponceau S Staining Solution for 10 min at room temperature. Then it was imaged through Biorad ChemiDoc Touch Imaging System (Bio-Rad Laboratories, Inc., Hercules, CA, USA). This showed the total protein transferred to the blot in each sample, which were used for target protein expression normalization. The membrane was blocked with 5% BSA in Tris-buffered saline with Tween 20 and incubated with a primary antibody at 4 °C overnight. The primary antibodies contained anti-rabbit monoclonal NF-κB (65 kDa) antibody (1:1000) (Cell Signaling Technology, Danvers, MA, USA), anti-rabbit monoclonal phospho-NF-κB (65 kDa) antibody (1:1000) (Cell Signaling Technology, Danvers, MA, USA), anti-rabbit recombinant monoclonal ERK1/2 (43 kDa) antibody (1:1000) (Selleckchem, Houston, TX, USA), anti-rabbit recombinant monoclonal phospho-ERK1/2 (43 kDa) antibody (1:1000) (Selleckchem, Houston, TX, USA), anti-rabbit monoclonal p38 (40 kDa) antibody (1:1000) (Cell Signaling Technology, Danvers, MA, USA), anti-rabbit monoclonal phospho-p38 (40 kDa) antibody (1:1000) (Cell Signaling Technology, Danvers, MA, USA), anti-mouse monoclonal STAT3 (79 kDa) antibody (1:1000) (Cell Signaling Technology, Danvers, MA, USA), and anti-rabbit polyclonal phospho-STAT3 (79 kDa) antibody (1:1000) (Cell Signaling Technology, Danvers, MA, USA). A goat anti-rabbit or mouse lgG (H + L) secondary antibody (1:8000) was used to incubate the membrane at room temperature for 1 h followed by incubation for 1 min with clarity western ECL substrate (Bio-Rad Laboratories, Inc., Des Plaines, IL, USA). The blot was then imaged in molecular imager. The intensity of the blot band was quantified and analyzed by Image Lab 6.0.1 (Bio-Rad Laboratories, Inc., Hercules, CA, USA). There were 3 samples in each group. Among all groups, three wells from a 6-well plate were pooled as a single sample to have sufficient amounts of protein for running Western blot assessments.

## 3. Statistical Analysis

All results are showed as mean values ± S.E.M. with 4–6 samples in per group. All biomolecule abundance differences between healthy and MetS groups were determined by one-way ANOVA with Tukey post hoc analysis; *p* < 0.05. Comparisons of cell viability, cellular association of NPs, and inflammatory responses were also statistically assessed using one-way ANOVAs with Tukey post hoc analysis between groups using disease (health or MetS) as the factor, and a *p* < 0.05 was considered to be statistically significant. All statistical analysis were processed in GraghPad Prism 9 software (GraphPad, San Diego, CA, USA).

## 4. Results

### 4.1. Mouse Model Characterization

High-fat diet demonstrated to increase the body weight of C57B6J mice compared to the healthy diet (Table 1). Moreover, total cholesterol, HDL, and LDL/VLDL levels in serum were higher in MetS mice fed with high-fat diet compared to the healthy mice (Table 1).

### 4.2. Characterization of Fe_3_O_4_ NPs

Hydrodynamic size, polydispersity index, and zeta potential of Fe_3_O_4_ NPs were characterized without or with healthy or MetS BALF BCs (Table 2). Hydrodynamic size was determined to be increased following addition of the BCs (Table 2). Addition of the MetS BC resulted in a larger hydrodynamic size compared to the healthy BC. The polydispersity of the NPs was increased following addition of both BCs (Table 2). Previous evaluations have demonstrated similar alterations in NP properties [22,29,31,41]. Specifically, we previously visualized, via transmission electron microscopy, biomolecules coating silver nanoparticles and demonstrated alterations in hydrodynamic size were primarily associated with changes in NP agglomeration [42]. Fe_3_O_4_ NP zeta potential was reduced following addition of both BCs with the change being more evident by addition of the MetS BC (Table 2).

### 4.3. Protein Components Identification and Relative Quantification between BCs

**Figure 1:**
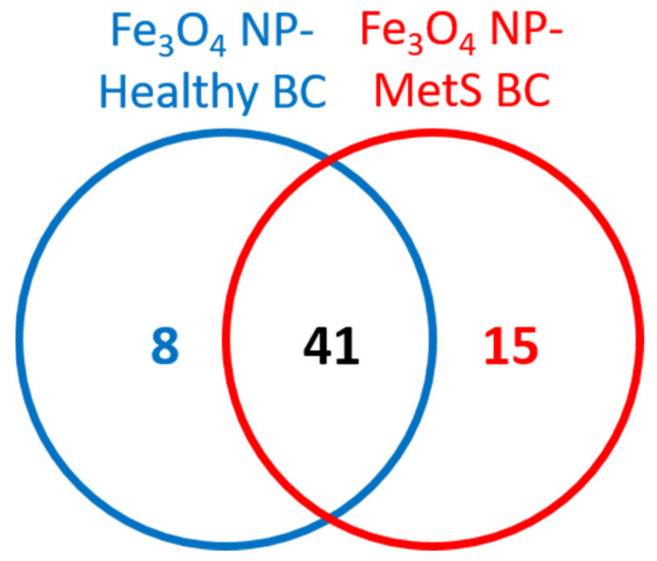
Comparison of BC composition between healthy and MetS conditions. Proteins identified as present in healthy BC were compared to proteins within the BC formed from MetS BALF. Venn diagrams were used to illustrate the number of unique and common proteins. A comprehensive list of these proteins and relative abundance of all proteins identified in each BC can be found in Table S1.

After the incubation in BALF Fe_3_O_4_ NPs formed unique BCs consisting of differential proteins as well as alterations in protein abundance. Specifically, 49 proteins were identified in the healthy BC, while 56 proteins were found in the MetS BC (Figure 1 and Table S1). Of these identified proteins, 41 were shared by both BCs (Figure 1). For example, these included tubulin alpha-1B chain, calpain small subunit 1, myosin light polypeptide 6, protein phosphatase 1 regulatory subunit 12A, and apolipoprotein A-IV. In healthy BALF, Fe_3_O_4_ NPs adsorbed 8 unique proteins, including Ig lambda-3 chain C region, F-box/LRR-repeat protein 12, histidine-rich glycoprotein dipeptidyl peptidase 1, dipeptidyl peptidase 1, mitogen-activated protein kinase 5, apolipoprotein A-I, dihydrolipoyl dehydrogenase, and chloride intracellular channel protein 5. However, incubation in MetS BALF, resulted in the accumulation of 15 distinct proteins, including alpha-enolase, coiled-coil domain-containing 146, exocyst complex component 6B, retinal dehydrogenase 1, selenium-binding protein 1, myosin-14, Heat shock protein HSP 90-beta, cytochrome b5, cytochrome P450 2F2, chitinase-3-like protein 1, bicaudal D-related protein 1, proteasome subunit beta type-6, pigment epithelium-derived factor, apolipoprotein C-III, and protease, serine 1 (trypsin 1).

**Figure 2:**
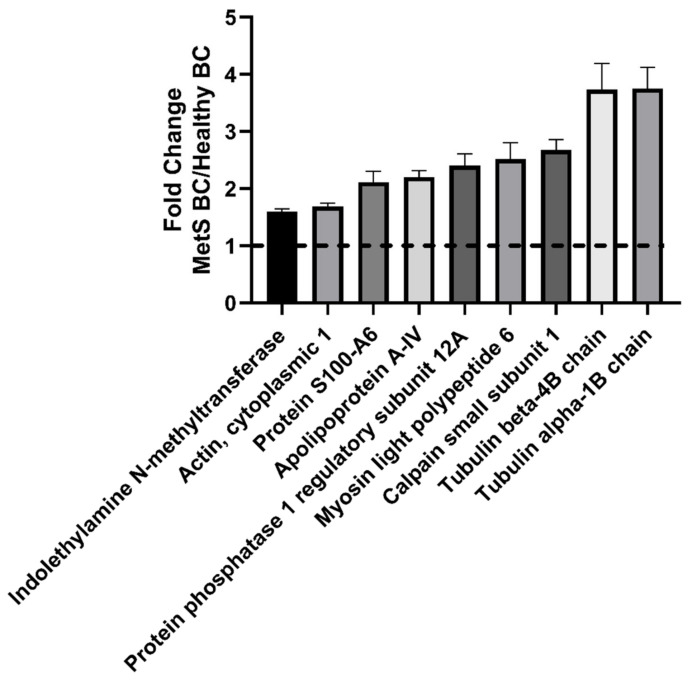
Relative abundance comparison of selected proteins found in healthy and MetS BCs. A value larger than 1 denotes a higher abundance in MetS BC compared to the healthy BC whereas a value less than 1 denotes lower abundance in the MetS BC compared to the healthy BC. All proteins depicted in the figure are significantly different between BCs based on *p* < 0.05 (n = 4/group). A comprehensive list of protein abundance differences can be found in Table S2.

Although numerous proteins were found in common between the healthy and MetS BC, differential abundance was determined (Table S2). Proteins determined to statistically differ between the two BCs in terms of abundance included: indolethylamine N-methyltransferase, actin, cytoplasmic 1, protein S100-A6, apolipoprotein A-IV, protein phosphatase 1 regulatory subunit 12A, myosin light polypeptide 6, calpain small subunit 1, tubulin beta-4B chain, and tubulin alpha-1B chain among all proteins in common (Figure 2). All significantly different proteins were more abundant in the MetS BC compared to the healthy BC.

### 4.4. Lipid Identification and Relative Quantification between BCs

**Figure 3:**
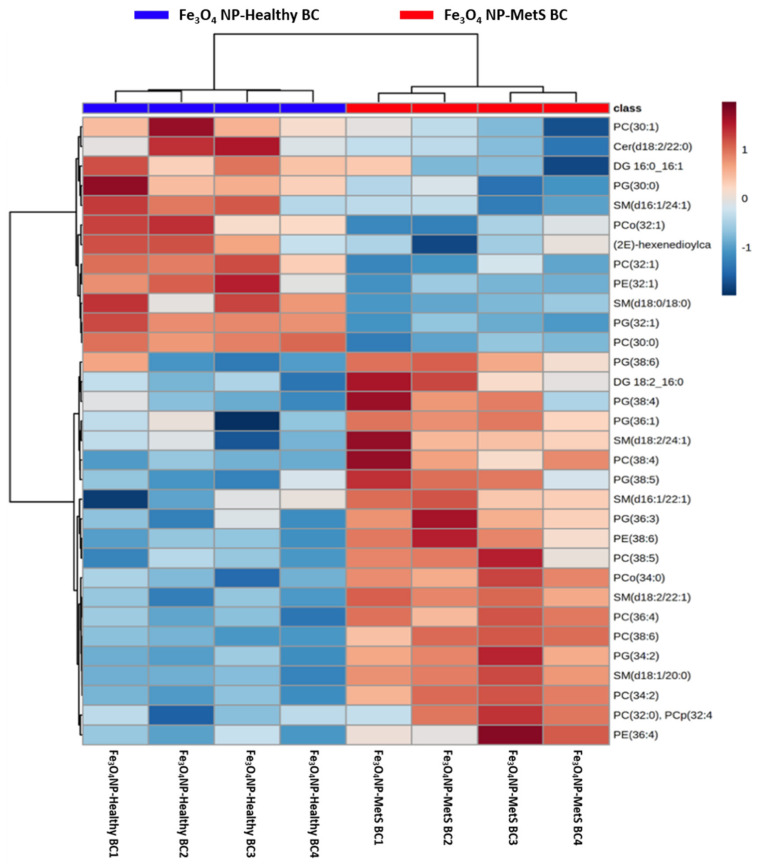
Relative abundance of lipids associated with healthy and MetS BCs. Lipids identified as present within the BCs in healthy and MetS conditions were compared based on their abundance (*p* < 0.05, n = 4/group). A heatmap provides intuitive visualization of differences between two BCs. Each colored cell on the map corresponds to a relative abundance of each lipid in the sample, with samples in rows and lipids in columns. Red denotes a higher concentration of the lipid in the sample, while blue denotes less lipid found in the sample compared to mean value of all samples from both groups. A comprehensive list of relative abundance differences for all lipids in BCs can be found in Table S3.

**Figure 4:**
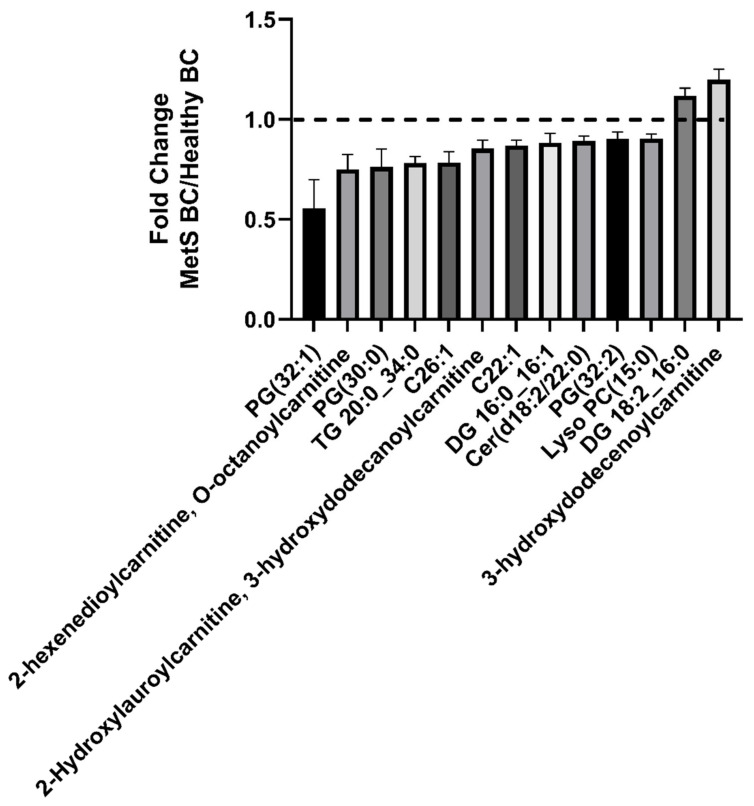
Fold change of selected lipids between the healthy and MetS BCs. Up/down regulation of selected lipids between each BCs are depicted as fold changes. Fold change was calculated based on the mean value of relative abundance of all samples from the same group. Values larger than 1 indicate the lipid was more abundant in MetS BC, while values less than 1 indicate that the lipid was more abundant in healthy BCs (*p* < 0.05, n = 4/group).

Characterization of the lipid components of the Fe_3_O_4_ NP BALF BCs included the screening of 1524 MRMs related to ceramides, phosphatidylcholines and sphingomyelins, phosphatidylethanolamines, phosphatidylglycerols, phosphatidylinositols, phosphatidylserines, cholesteryl esters, triacylglycerols (triglycerides), and the metabolite class of acyl-carnitines. Only 219 lipids were considered for the lipid profile statistical assessment (determined by being more than 30% above the blank sample intensity) (Table S3). Both BALF BCs were found to adsorb these 219 lipids in common, however, 32 were observed to significantly differ between the healthy and MetS BC (Figure 3). These lipids included PC(30:1), Cer(d18:2/22:0), DG 16:0_16:1, PG(30:0), SM(d16:/24:1), PG(38:6), DG 18:2_16:0, PG(38:4, PG(36:1), and SM(d18:2/24:1). Out of these 32 differentially abundant lipids, 20 were determined to be at higher abundance while 12 were less abundant in the MetS BC compared to the healthy BC. 13 lipids shown in Figure 4 were found to be the most significant. Of the 13 lipids demonstrating the largest fold changes between BCs, only DG 18:2_16:0 and 3-hydroxydodecenoylcarnitine were found to be more abundant in MetS BC compared to the healthy BC. The other 11 lipids demonstrated less abundance in the MetS BALF BC compared to the healthy BC. These included PG(32:1), 2-hexenedioylcarnitine, PG(30:0), TG 20:0_34:0, C26:1, 2-Hydroxylauroylcarnitine, C22:1, DG 16:0_16:1, Cer(d18:2/22:0), PG(32:2), and Lyso PC(15:0) (Figure 4).

### 4.5. BC-Induced Cell Viability in Macrophages

**Figure 5:**
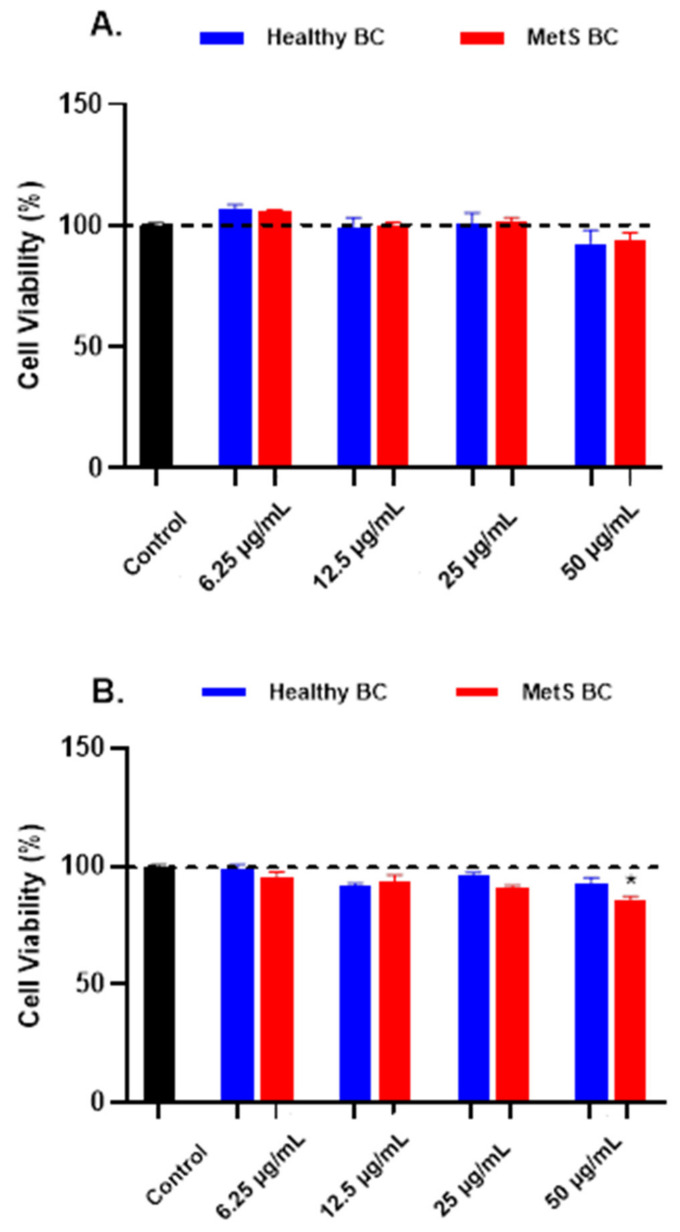
Cell viability following 1 h or 24 h NP with healthy or MetS BCs. Macrophages were exposed to Fe_3_O_4_ NPs with BCs at concentrations of 0, 6.25, 12.5, 25, or 50 μg/mL for 1 h (**A**) or 24 h (**B**), and cell viability was assessed through Thiazolyl Blue Tetrazolium Bromide (MTT) cell proliferation assay. Untreated cells in SFM were used as controls for the MTT assay. * Denotes statistical significance compared to the expression of control group (n = 4/group and *p* < 0.05).

A macrophage cell line was exposed to Fe_3_O_4_ NPs with BCs at concentrations of 6.25, 12.5, 25, and 50 μg/mL for 1 h or 24 h in serum-free medium (SFM) to determine differential cellular responses due to addition of the BC. No overt cytotoxicity (cell death > 20%) was observed following a 1 h (Figure 5A) or 24 h (Figure 5B) exposure. Only macrophages exposed to Fe_3_O_4_ NPs with a MetS BALF BC at 50 μg/mL, demonstrated reduced viability compared to controls (Figure 5B).

### 4.6. Influence of BCs on the Cellular Associations of Fe_3_O_4_ NPs

**Figure 6:**
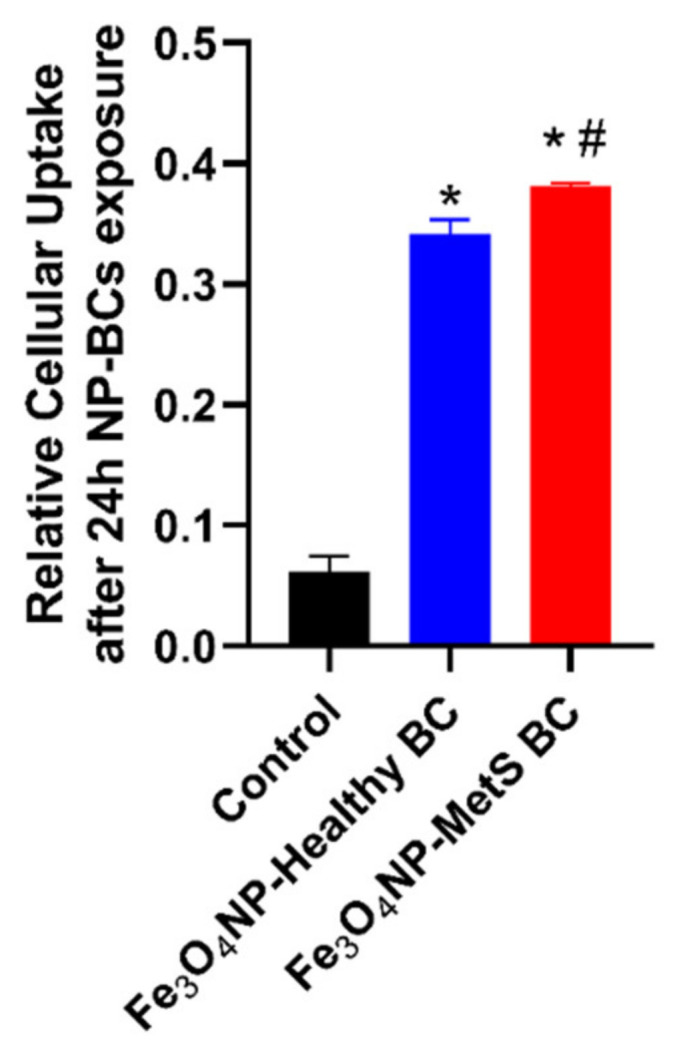
Measurement of cellular uptake of Fe_3_O_4_ NP with BCs by XRF analyzer. Macrophages were exposed to 25 μg/mL Fe_3_O_4_ NPs with BCs for 24 h (n = 3/group). Cellular uptake of Fe_3_O_4_ NPs was compared with untreated cells in SFM used as controls for the XRF analysis. * Denotes statistical significance compared to the expression of control group, ^#^ denotes statistical significance compared to the Fe_3_O_4_ NPs with a healthy BC (*p* < 0.05).

BC-induced alterations in Fe_3_O_4_ NPs-cellular association were demonstrated via XRF analysis. Cells were harvested after a 24 h exposure to 25 μg/mL Fe_3_O_4_ NPs. Exposure to Fe_3_O_4_ NPs resulted in increased cellular associated iron levels after normalization to total protein concentration (Figure 6). Fe_3_O_4_ NPs with a MetS BALF BC enhanced cellular associated iron levels compared to Fe_3_O_4_ NPs with a healthy BC (Figure 6).

**Figure 7:**
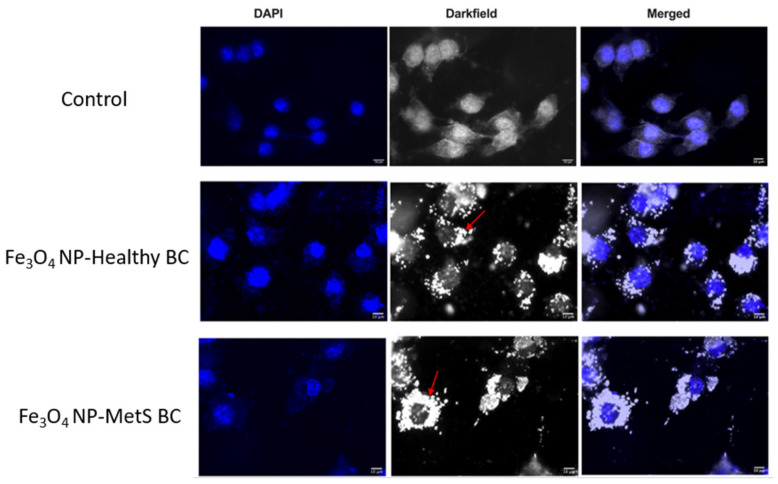
Assessment of cellular association of Fe_3_O_4_ NPs by dark-field microscopy. Representative images of internalized Fe_3_O_4_ NPs in control, healthy BCs, and MetS BCs groups. Bright dots labeled with red arrows indicate distribution of Fe_3_O_4_ NPs in macrophages.

To qualitatively confirm BC-induced alterations in cellular associations of Fe_3_O_4_ NPs, macrophages were assessed via dark field microscopy following 24 h exposure to Fe_3_O_4_ NPs with healthy and MetS BCs at a concentration of 25 μg/mL. This evaluation fluorescently labelled the nuclei of macrophages enabling images to be focused on the nuclear plane. Macrophages were validated to associate Fe_3_O_4_ NPs following 24 h exposure (Figure 7). Macrophages exposed to Fe_3_O_4_ NPs with a MetS BALF BC were found to increasingly associate with macrophages compared to Fe_3_O_4_ NPs with a healthy BALF BC (Figure 7).

### 4.7. Evaluation of BC-Induced Differential Cellular Inflammatory Response

**Figure 8:**
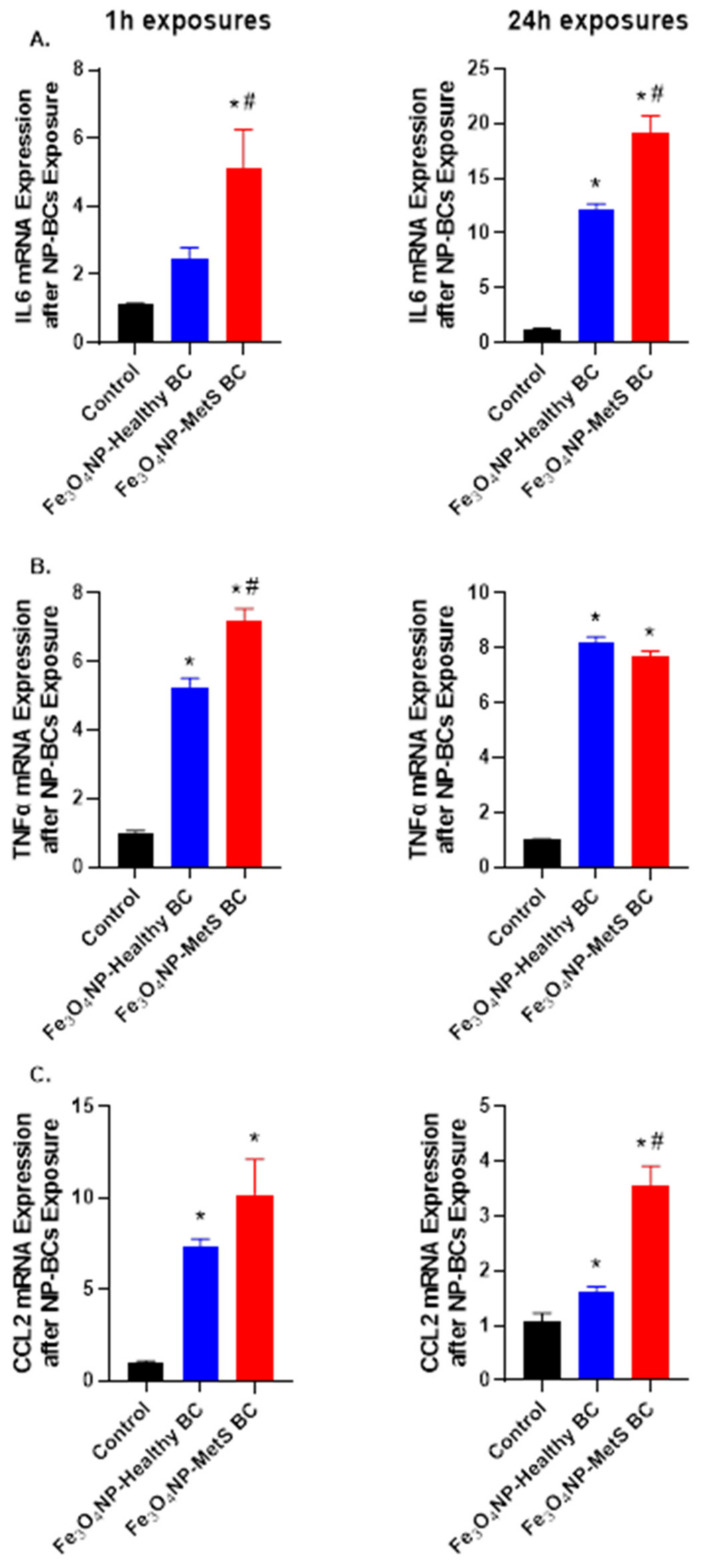
Alterations in inflammatory gene expression due to variations in the NP-BC. Changes in gene expression following exposure to Fe_3_O_4_ NPs with a healthy or MetS BC. Expression of mRNA was assessed relative to serum-free media control cells (untreated). Cells were exposed to 25 μg/mL of Fe_3_O_4_ NPs with BCs for 1 h or 24 h (n = 5/group). Gene expression of *IL6* (**A**), *TNFα* (**B**), and *CCL2* (**C**), and *GAPDH* (housekeeping gene) was assessed through RT real-time PCR to evaluate the inflammatory responses due to BCs. * Denotes statistical significance compared to the expression of control group, ^#^ denotes statistical significance compared to the Fe_3_O_4_ NPs with a healthy BC (*p* < 0.05).

Inflammatory gene expression alterations were examined in macrophages following exposure to 25 μg/mL of Fe_3_O_4_ NPs with BCs for 1 h or 24 h (Figure 8). Following a 1 h exposure, gene expression of IL-6 was increased following exposure to Fe_3_O_4_ NPs with a MetS BC. At 24 h exposure to Fe_3_O_4_ NPs with both BCs up-regulated IL-6 gene expression with the MetS suggesting an exacerbated response (Figure 8A). Macrophage TNF-α gene expression was elevated at 1 h following exposure to Fe_3_O_4_ NPs with healthy and MetS BCs. Fe_3_O_4_ NPs with a MetS BC showed an enhanced induction of TNF-α gene expression compared to Fe_3_O_4_ NPs with a healthy BC at 1 h. The 24 h exposure to Fe_3_O_4_ NPs with both BCs resulted in a similar induction of TNF-α gene expression by macrophages (Figure 8B). Lastly, CCL2 gene expression was assessed and demonstrated upregulation following a 1 h exposure to Fe_3_O_4_ NPs with both BCs which was enhanced due to the MetS BC. At 24 h CCL2 gene expression supported similar pattern of response as compared to the 1 h time point (Figure 8C).

### 4.8. Quantification and Determination of Protein Expression

**Figure 9:**
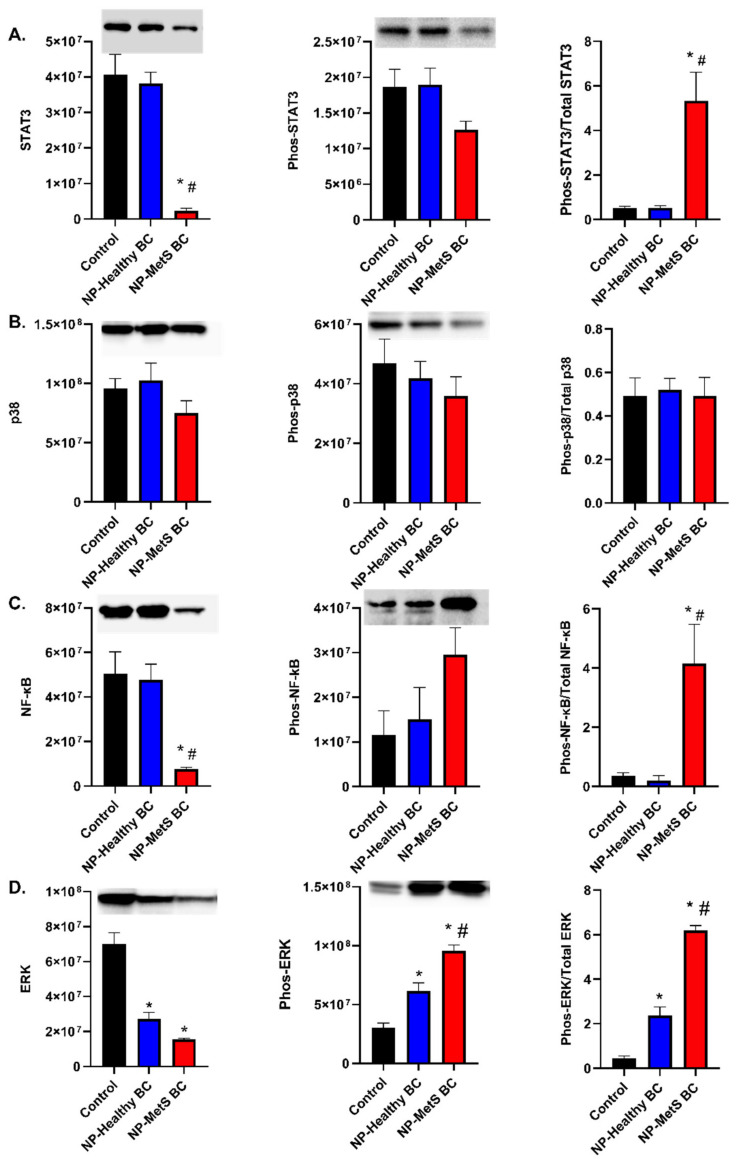
Inflammatory signaling after a 1 h exposure to NPs with MetS or healthy BCs. Changes in protein level following exposure to Fe_3_O_4_ NPs with a healthy or MetS BC. Cells were exposed to 25 μg/mL of Fe_3_O_4_ NPs with BCs for 1 h (n = 3/group). Activation of STAT3 (**A**), P38 (**B**), NF-κB (**C**), and ERK (**D**) pathways were assessed through Western blot to evaluate MetS-related inflammatory signaling pathways following NPs exposures. * Denotes statistical significance compared to the expression of control group, ^#^ denotes statistical significance compared to the Fe_3_O_4_ NPs with a healthy BC (*p* < 0.05). Quantification of each protein was normalized to the intensity of total protein (blots found in Supplementary Materials Figure S1a–d).

Inflammatory signaling was evaluated in macrophages following 1 h or 24 h exposure to Fe_3_O_4_ NPs with BALF BCs at a concentration of 25 μg/mL. Following a 1 h exposure, total STAT3 significantly decreased following exposure to NPs with a MetS BC, which increased of the ratio of phosphorylated STAT3 to total STAT3 (Figure 9A). Exposure to NPs with either BCs did not alter total p38 or phosphorylated p38 (Figure 9B). Decreased expression of total NF-κB was observed resulting in an increase of the ratio of phosphorylated NF-κB to total NF-κB (Figure 9C). Total ERK was significantly decreased following exposure to both BCs compared to the control group. Phosphorylated ERK demonstrated significant increases following exposure to NPs with either BC. Specifically, exposure to NPs with a MetS BC enhanced phosphorylation of ERK compared the healthy BC (Figure 9D).

**Figure 10:**
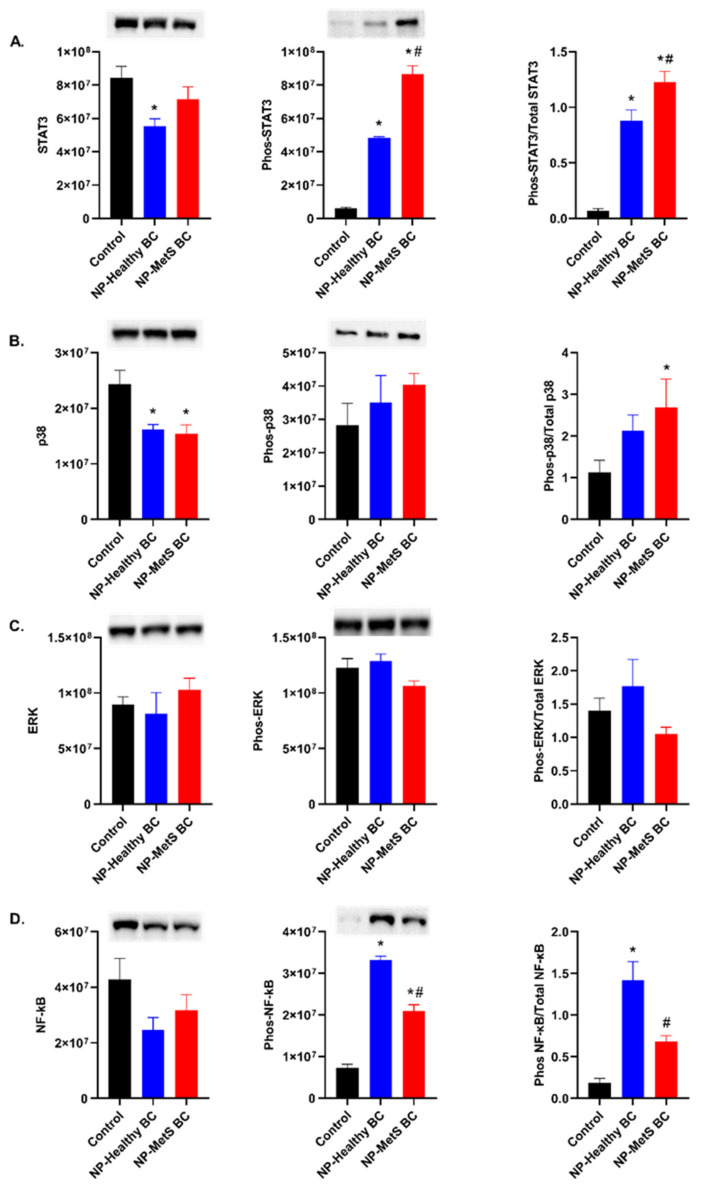
Inflammatory signaling after a 24 h exposure to NPs with MetS or healthy BCs. Changes in protein level following exposure to Fe_3_O_4_ NPs with a healthy or MetS BC. Cells were exposed to 25 μg/mL of Fe_3_O_4_ NPs with BCs for 24 h (n = 3/group). Activation of STAT3 (**A**), P38 (**B**), NF-κB (**C**), and ERK (**D**) pathways were assessed through Western blot to evaluate MetS-related inflammatory signaling pathways following NPs exposures. * Denotes statistical significance compared to the expression of control group, ^#^ denotes statistical significance compared to the Fe_3_O_4_ NPs with a healthy BC (*p* < 0.05). Quantification of each protein was normalized to the intensity of total protein (blots found in Supplementary Materials Figure S1e–h).

At the 24 h time point, exposure to Fe_3_O_4_ NPs with healthy BCs significantly decreased expression of total STAT3 compared to the control group (Figure 10A). Exposure to both BCs significantly increased levels of phosphorylated STAT3 compared to controls. These alterations resulted in an increased ratio of phosphorylated to total STAT3 due to exposure which was enhanced due to the MetS BC. Total p38 was significantly decreased following exposure to Fe_3_O_4_ NPs with both BCs. No significant differences in phosphorylation of p38 were observed due to exposures. However, the ratio of phosphorylated p38 to total p38 was determined to be significantly increased in macrophages exposed to Fe_3_O_4_ NPs with MetS BCs (Figure 10B). No alterations in total or phosphorylated ERK were determined for either exposure group compared to controls (Figure 10C). Total NF-κB levels were reduced following exposure to Fe_3_O_4_ NPs with healthy or MetS BCs as compared to the control group. Phosphorylation of NF-κB was found to be significantly increased following exposure to Fe_3_O_4_ NPs with both BCs. However, the healthy BC was determined to induce a greater amount of phosphorylated NF-κB compared to the MetS BC. The ratio of phosphorylated NF-κB to total NF-κB was elevated for both exposure groups, however more so for Fe_3_O_4_ NPs with a healthy BC. (Figure 10D).

## 5. Discussion

Inhalation of nanosized particulate matter and engineered nanoparticles (NPs) results in pulmonary inflammation and toxicity. Evaluations of biocorona formed on the surface of NPs containing different biological molecules such as proteins after contacting with biological fluids have been confirmed to affect cellular uptake, intracellular NP location, and cell responses [43]. Individuals suffering from chronic underlying disease have demonstrated exacerbated health effects following inhalation exposures. Currently, the mechanisms of toxicity contributing to these exacerbated responses are unelucidated, impeding the development of treatments and regulatory standards to protect the most susceptible subpopulations. Metabolic syndrome (MetS) is a prevalent and expanding disease state and individuals suffering from it have demonstrated enhanced adverse health effects following inhalation exposures. MetS and other chronic diseases are associated with modifications to the biological *milieu* which may alter the initial interactions between NPs and biomolecules resulting in the formation of unique NP-biocoronas (NP-BCs). These distinct BCs may facilitate exacerbated pulmonary immune responses contributing to variations in toxicity and inflammation observed in individuals with chronic diseases following inhalation exposures. In our current study, isolated bronchoalveolar lavage fluid samples from healthy and MetS mouse models were utilized to evaluate NP-BC formation and its consequences related to macrophage interactions and inflammatory signaling. Our results demonstrated addition of healthy and MetS BCs distinctly altered NP properties. Further, distinct protein and lipid contents were identified to associate with Fe_3_O_4_ NPs in healthy or MetS states. Addition of the MetS BC enhanced macrophage uptake compared to of NPs with a healthy BC. While both BCs induced inflammatory gene expression, the MetS BC was determined to exacerbate expression of *IL6* and *CCL2* compared to the healthy BC. STAT3, NF-κB, and ERK pathways were upregulated and likely facilitate the enhanced inflammatory responses observed following exposure to the MetS-BC. Overall, our results suggest MetS causes variations in the formation of the BC which may contribute to exacerbated pulmonary toxicity and inflammation following inhalation of NPs. 

Previous studies have demonstrated disease-induced variations in biological environments such as hyperlipidemia influenced the formation of NP-BCs and altered cellular responses [29,40]. Additionally, NP-protein complexes abundant with Apo A-I were able to interact with certain scavenger receptors (SRs) such as SR-B1 which are expressed on the surface of many cells including macrophages. Increased uptake of NPs was observed with elevated expression of SR-B1, suggesting a possible mechanism of interaction between protein contents contained in the corona and SRs on the surface of cells [44]. Additionally, a BC consisting of only LDL was determined to enhance the cellular uptake of NPs via SRs as well. Macrophages exposed to the Fe_3_O_4_ NPs with an LDL-BC demonstrated increased TNF-α mRNA expression compared to a BC formed in fetal bovine serum [29]. This suggested that distinct components within the BC can facilitate cellular interactions and inflammatory responses. To date, formation of BCs has been principally investigated in serum or plasma to examine intravenous routes of exposure. However, inhalation is the primary route of Fe_3_O_4_ NPs exposure, especially in environmental and occupational settings. A better understanding of the pulmonary NP-BC and its effects is therefore necessary to examine initial interactions occurring after inhalation which may be responsible for acute cell responses. Further, toxicology assessments often utilize healthy models to assess NP safety, which may not be applicable to susceptible groups. A previous study described the NP-surface corona that formed following incubation in human BALF collected from patients with pulmonary alveolar proteinosis. This assessment determined there was a potential for distinct cellular effects due to differential binding of surfactant-associated proteins to the NP surface. For example, some proteins such as surfactant proteins A and D associating with NPs demonstrated increased abundance due to disease and were able to interact with cells influencing NP phagocytosis [45].

The current study examined biological contents including proteins and lipids associated with the surface of all Fe_3_O_4_ NPs in both healthy and MetS states. The utilization of both approaches provides for a comprehensively assessment of NP-BC formation. Differing numbers of unique proteins associated with Fe_3_O_4_ NPs in healthy and MetS conditions. When incubated in MetS BALF, 15 unique proteins were absorbed on Fe_3_O_4_ NPs, indicating an underlying disease state can influence the association of proteins and NPs. Among these unique proteins, some were expected to be identified in MetS BC, as they are confirmed to relate to MetS. For example, glycolytic enzyme alpha-enolase and apolipoprotein c-III were uniquely present in the MetS BC and are elevated in metabolic diseases [46,47]. Following incubation in the BALF, NPs are more likely to associate these proteins on their surface due to their increased abundance in individuals suffering from MetS. Some proteins that were unique in the MetS BC cannot be directly explained by MetS-related alterations, however, they are known to be present in the respiratory system and elevated in inflammatory diseases. For example, alpha-enolase, chitinase-3-like protein 1, and pigment epithelium-derived factor are involved in inflammatory signaling, autoimmune diseases, and insulin resistance [46,48,49,50]. MetS is associated with the development of insulin resistance and underlying inflammation. Our previous studies have not demonstrated differential levels of circulating insulin or inflammatory markers within the lung at this stage in MetS disease development compared to healthy mice [20,21]. Disease-associated alterations in these proteins may occur prior changes in clinical markers of insulin resistance and inflammation. Among the 41 common proteins, some proteins were more abundant in the MetS BC compared to the healthy BC. Specifically, apolipoprotein A4 was determined to be a component in both BCs; however, it was more abundant in MetS BC. This increased abundance is expected due to apolipoprotein A4 being involved in metabolic diseases such as obesity [51]. Furthermore, more abundant in the MetS BC was myosin light polypeptide 6 which is regulated by myosin light chain kinase. Imbalance of this kinase in airway lining fluid due to diseases is associated with pulmonary disorders [52].

Lipid components also demonstrated differences in healthy and MetS BCs. Among 32 lipids showing significance differences, 20 were more abundant in the MetS BC compared to the healthy BC. Several phosphatidylcholine species with a relatively high total carbon number were found to relate to common chronic diseases, such as PC(38:4) and PC(34:2). The level of PC(38:4) has been observed to be increased in individuals with premature coronary heart disease (CHD) or MetS whereas PC(34:2) is also elevated in SHD patients and slightly higher in MetS patients [53]. SM(d18:1/20:0) is a sphingolipid and demonstrated higher abundance in the MetS BC compared to the healthy BC. Sphingolipids function as a component of cellular membranes and participate in inflammation [54]. Further research illustrates emerging roles of sphingolipids in cellular signaling, as well as serving as an important constituent of the lung fluid environment [55]. Our assessment also revealed significant increases in 3-hydroxydodecenoylcarnitine and DG18:2_16:0 in the MetS BC. 3-hydroxydodecenoylcarnitine is an acyl-carnitine, and is found to relate to inflammatory changes in adipose tissue in animal models associated with obesity [56]. DG18:2_16:0 is a diacylglycerol functioning as a physiological activator of protein kinase C (PKC) by facilitating translocation of PKC from the cytosol to the plasma membrane and diacylglycerol-induced signaling may contribute to insulin resistance [57]. Individuals with MetS are more likely to have altered lipid levels in the airway fluid which are known to be elevated in MetS patients. The variable lipid levels result in modification of NP-BCs formation and subsequent cellular responses to insulin suppressing the ability to use glucose from blood for energy. Overall, this assessment of BC compositions suggests the MetS condition alters both the protein and lipid contents of BALF which can affect interactions with NPs. The differences in protein and lipid compositions associated with NPs in MetS state may modulate susceptibility following NP exposure.

Following inhalation, NPs are predominately located within alveolar macrophages demonstrating that they are a primary cell type interacting with NPs following exposures [58]. Therefore, we chose to utilize the macrophage as the cellular model to determine the biological impact of disease-related variations in the NP-BC. Macrophages function to initiate the immune response within the lung responding to inhaled insults. Specifically, they function as the primary innate immune cell responsible for identification and phagocytosis of foreign substances mediating digestion and elimination. During these immune processes macrophages produce cytokines and chemokines regulating the inflammatory response within the lung [59]. Scavenger receptors (SRs) such as SR-A1, SR-A6, and SR-B1 on macrophage cell surface may interact with NPs or the biomolecular corona of NPs, mediating the cellular uptake [29,44,60,61]. Our study demonstrated the MetS BC increased the cellular association of NPs in macrophages compared to a healthy BC. This result was consistent with the images collected from dark field microscopy. More cellular association of Fe_3_O_4_ NPs indicated increased interactions between NPs and macrophages which may also contribute to differential biological impact in MetS.

Assessment of BC-induced alterations in gene expression revealed exacerbated inflammatory responses due to MetS. Some protein and lipid contents identified in the MetS BC are known to be involved in regulation of inflammation. For example, alpha-enolase, chitinase-3-like protein 1, pigment epithelium-derived factor, SM(d18:1/20:0), and 3-hydroxydodecenoylcarnitine were increased in the MetS BC and facilitate inflammation. Specifically, cell-surface expression of alpha-enolase is increased on macrophages isolated from rheumatoid arthritis patients. Moreover, antibodies against it can stimulate macrophages to produce more proinflammatory mediators, such as TNF-α and IL-1 α/β [62]. Apolipoprotein C-III was also identified to be increased in the MetS BC compared to the healthy BC. It is not only a MetS-related protein but also able to induce inflammation and organ damage [50], and may facilitate exacerbated inflammatory responses. Increases of *IL6* and *CCL2* gene expression were observed following exposure to both NP-BCs, however, the MetS BC was determined to stimulate exacerbated responses. Increases in gene expression of IL-6, TNFα, and CCL2 showed 1 h after exposure to NPs with MetS BCs corresponded with changes observed in specific proteins within the MAP kinase, Jak/Stat, and NF-κB signaling pathways. These increases are likely driven by an addition of MetS BCs on NPs suggesting enhanced inflammatory responses were induced after 1 h via activation STAT3, NF-κB, and ERK pathways but not the p38 pathway due to MetS BCs. Previous studies have demonstrated activation of p38 or NF-κB pathways can increase transcription of *CCL2* [63,64,65]. Further, increased STAT3 phosphorylation levels can trigger *CCL2* transcription to enhance macrophag e recruitment [66]. *CCL2* demonstrated the largest fold increase following 1 h exposure to NPs with MetS BCs and is likely to be related to activation of STAT3, ERK, and NF-κB pathways. Previously, upregulation of *CCL2* transcription was determined to be mediated through the ERK pathway following cationic liposome stimulation of dendritic cells [67]. At 24 h, enhanced gene expressions of *IL6* and *CCL2* as well as phosphorylation of STAT3 and p38 was observed in response MetS BC exposure. These significant changes may be induced in response to the MetS BC as well as the previous release of cytokines or chemokines. Specifically, *IL6* was found to be the most different between healthy and MetS BCs groups. A previous study showed that *IL6* can mediate inflammation and activation of STAT3 via JAK [68]. Additionally, abnormally prolonged phosphorylation of STAT3 plays a role in inflammation where the level of *IL6* secretion is often elevated. Thus, after 24 h *IL6* may be able to mediate activation of STAT3, and p38 inflammatory pathways subsequently enhancing macrophage to release chemokines, such as *CCL2* and exacerbating inflammation. At the same time, p38 pathway is determined to mediate inflammatory responses [69]. An activation of this pathway can also be responsible for exacerbated inflammation and subsequent susceptibility. MetS BC appears to stimulate distinct pathways involved in inflammation more robustly than the healthy BC. Elevated activation of STAT3 pathway due to an addition of MetS BCs may exacerbate inflammatory responses and result in susceptibility to inhaled exposures.

Overall, our study demonstrates the potential for distinct BCs form on the surface of NPs in disease states within the lung potentially mediating acute pulmonary inflammatory responses and susceptibility. In the future, we aim to investigate the contribution of specific inflammatory pathways via pharmacological inhibition of specific targets including STAT3, NF-κB, or ERK. Additionally, we will identify distinct BC component interactions with immune cell surface receptors involved in particle recognition and inflammation. Specifically, this assessment will focus on components such as apolipoproteins and sphingomyelins as well as scavenger receptors that may mediate cell interactions. Furthermore, assessments will incorporate other pulmonary cells such as alveolar epithelial cells to determine their interactions with the NP-BC and contribution to differential toxicity following inhalation exposure. Our current assessment was comprehensive as it evaluated differential protein and lipid contents of the BALF BC in two models. The differential initial interactions within the lung may govern subsequent cellular responses and contribute to enhanced inflammation to inhaled NPs exposures in susceptible subpopulations such as MetS.

## Figures and Tables

**Table 1:** Characterization of serum lipid levels in healthy and MetS mouse models.

Mouse Model	Body Weight (g)	Serum Total Cholesterol (mg/dL)	Serum LDL/VLDL (mg/dL)	Serum HDL (mg/dL)
Healthy	26.2 ± 0.4	91.5 ± 3.6	10.9 ± 0.5	84.0 ± 1.0
MetS	48.7 ± 0.4 *	193.7 ± 3.6 *	21.2 ± 0.4 *	140.4 ± 1.3 *

Data is expressed as mean ± SEM, n = 5/group. *: *p* < 0.05, as compared to the healthy mouse model.

**Table 2:** Fe_3_O_4_ NPs-biocorona characterization.

Nanoparticle—BC	Hydrodynamic Size (nm)	Polydispersity Index	Zeta Potential (mV)
Fe_3_O_4_	46.6 ± 1.0	0.14 ± 0.01	27.4 ± 1.1
Fe_3_O_4_-Healthy BC	144.5 ± 1.8 *	0.42 ± 0.01 *	25.8 ± 0.9
Fe_3_O_4_-MetS BC	218.2 ± 1.8 *^,#^	0.49 ± 0.02 *^,#^	23.8 ± 0.7 *

Data is expressed as mean ± SD, n = 3/group. *: as compared to NPs without a BC, *p* < 0.05, ^#^: as compared to the Fe_3_O_4_ NPs with a healthy BC, *p* < 0.05.

## Data Availability

All the raw LC-MS/MS proteomic data are deposited in the MassIVE data repository (massive.ucsd.edu/ accessed on 28 April 2022) with ID MSV000089322. Additionally, all raw lipid profiling data have been submitted to purr Purdue.

## Supplementary Materials

The following supporting information can be downloaded at: https://www.mdpi.com/article/10.3390/nano12122022/s1, Table S1: Comprehensive list of proteins and relative abundance of all proteins identified in each BC; Table S2: Proteins found to associate with Fe_3_O_4_ NPs in Healthy and MetS conditions; Table S3: Comprehensive list of relative abundance differences for all lipids in BCs; Figure S1: 1 h Total protein for (a) STAT3 & p-STAT3 (b) p38 & p-p38 (c) NF-κB & p-NF-κB (d) ERK & p-ERK. 24 h Total protein for (e) STAT3 & p-STAT3 (f) p38 & p-p38 (g) ERK & p-ERK (h) NF-κB & p-NF-κB.
