# Supplementary material for: Modulation of Pulmonary Toxicity in Metabolic Syndrome Due to Variations in Iron Oxide Nanoparticle-Biocorona Composition
Source: Nanomaterials (Basel). 2022 Jun 11;12(12):2022. doi: 10.3390/nano12122022 (PMC9230893; doi:10.3390/nano12122022)

Supplementary Materials

Modulation of Pulmonary Toxicity in Metabolic Syndrome Due to Variations in Iron Oxide Nanoparticle-Biocorona Composition

Li Xia [1], Saeed Alqahtani [1,2], Christina R. Ferreira [3], Uma K. Aryal [4,5], Katelyn Biggs [1] and Jonathan H. Shannahan [1,*]

[1] School of Health Sciences, College of Health and Human Sciences, Purdue University, West Lafayette, IN 47907, USA; xia104@purdue.edu (L,X,); salqaht@purdue.edu (S,A,); biggs23@purdue.edu (K,B,)
[2] Life Science and Environment Research Institute, King Abdulaziz City for Science and Technology (KACST), Riyadh 12354, Saudi Arabia
[3] Purdue Metabolite Profiling Facility, Purdue University, West Lafayette, IN 47907, USA; cferrei@purdue.edu
[4] Purdue Proteomics Facility, Bindley Bioscience Center, Purdue University, West Lafayette, IN 47907, USA; uaryal@purdue.edu
[5] Department of Comparative Pathobiology, Purdue University, West Lafayette, IN 47907, USA
* Correspondence: jshannah@purdue.edu

Table S1. Comprehensive list of proteins and relative abundance of all proteins identified in each BC.

Protein ID	Gene names	Protein names	Average Fe_3O_4 NP-Healthy BC	Average Fe_3O_4 NP-MetS BC	Fold Change Average Fe_3O_4 NP-MetS BC	p-value from Core Analysis
Q9DBR7	Ppp1r12a	Protein phosphatase 1 regulatory subunit 12A	3187175,0	7652625,0	2,4011	0,0033
P05213	Tuba1b;Tuba1c;Tuba4a	Tubulin alpha-1B chain;Tubulin alpha-1C chain;Tubulin alpha-4A chain	2434125,0	9131000,0	3,7512	0,0045
P68372	Tubb4b;Tubb4a;Tubb5;Tubb2b;Tubb2a	Tubulin beta-4B chain;Tubulin beta-4A chain;Tubulin beta-5 chain;Tubulin beta-2B chain;Tubulin beta-2A chain	2582655,0	9651800,0	3,7372	0,0047
O88456	Capns1	Calpain small subunit 1	1201787,5	3219575,0	2,6790	0,0105
P14069	S100a6	Protein S100-A6	9217425,0	19505500,0	2,1162	0,0178
P06728	Apoa4	Apolipoprotein A-IV	4175300,0	9194625,0	2,2021	0,0245
P60710	Actb;Actg1	Actin, cytoplasmic 1;Actin, cytoplasmic 1, N-terminally processed;Actin, cytoplasmic 2;Actin, cytoplasmic 2, N-terminally processed	29657250,0	50094000,0	1,6891	0,0295
Q60605	Myl6	Myosin light polypeptide 6	611782,5	1924900,0	2,5160	0,0313
P40936	Inmt	Indolethylamine N-methyltransferase	6734625,0	10728150,0	1,5930	0,0477
P09813	Apoa2	Apolipoprotein A-II;Proapolipoprotein A-II	10365900,0	16205250,0	1,5633	0,0545
P07309	Ttr	Transthyretin	2894125,0	2150600,0	0,7431	0,0598
P29788	Vtn	Vitronectin	169771,0	65805,3	0,2907	0,0658
Q00896	Serpina1c;Serpina1a	Alpha-1-antitrypsin 1-3;Alpha-1-antitrypsin 1-1	2290953,3	763240,0	0,3332	0,0663
Q6GT24	Prdx6	Peroxiredoxin-6	5251375,0	7082100,0	1,3486	0,0720
O08553	Dpysl2	Dihydropyrimidinase-related protein 2	503667,3	1349372,5	2,6791	0,0727

Q06890	Clu	Clusterin;Clusterin beta chain;Clusterin alpha chain	997750,0	635170,0	0,6366	0,0803
A0A0R4J0I1	Serpina3k;Serpina3m	Serine protease inhibitor A3K;Serine protease inhibitor A3M	3098000,0	1139855,0	0,3679	0,0951
P29699	Ahsg	Alpha-2-HS-glycoprotein	2809045,0	837717,5	0,2982	0,0962
P07724	Alb	Serum albumin	11311450,0	6749400,0	0,5967	0,1062
Q09XV5	Chd8	Chromodomain-helicase-DNA-binding protein 8	1464925,0	1521900,0	1,0389	0,1272
P16858	Gapdh;Gm3839	Glyceraldehyde-3-phosphate dehydrogenase	732212,5	1240837,5	1,6946	0,1277
P68134	Acta1;Actc1;Acta2;Actg2	Actin, alpha skeletal muscle;Actin, alpha cardiac muscle 1;Actin, aortic smooth muscle;Actin, gamma-enteric smooth muscle	530610,0	789837,5	1,4885	0,1348
P10126	Eef1a1;Eef1a2	Elongation factor 1-alpha 1;Elongation factor 1-alpha 2	693116,7	1093617,5	1,5778	0,1440
Q8BHL4	Gprc5a	Retinoic acid-induced protein 3	502403,3	272796,7	0,5430	0,1530
P08074	Cbr2	Carbonyl reductase [NADPH] 2	1895825,0	2863525,0	1,5104	0,1631
O09043	Napsa	Napsin-A	3905800,0	4698200,0	1,2029	0,2086
Q00897	Serpina1d	Alpha-1-antitrypsin 1-4	6662350,0	4884900,0	0,7332	0,2148
A8DUK4	Hbbt1; Hbb-b1; Hbb-bs; Hbb-b2	Hemoglobin subunit beta-1;Hemoglobin subunit beta-2	4589025,0	5977875,0	1,3026	0,2157
P01027	C3	Complement C3;Complement C3 beta chain;C3-beta-c;Complement C3 alpha chain;C3a anaphylatoxin;Acylation stimulating protein;Complement C3b alpha chain;Complement C3c alpha chain fragment 1;Complement C3dg fragment;Complement C3g fragment;Complement C3d fragment;Complement C3f fragment;Complement C3c alpha chain fragment 2	235347,5	172255,0	0,7319	0,2185
P52480	Pkm	Pyruvate kinase PKM	234572,5	508190,0	2,1665	0,2245
P13020	Gsn	Gelsolin	3748700,0	2873100,0	0,7664	0,2350
P50404	Sftpd	Pulmonary surfactant-associated protein D	15711025,0	12751000,0	0,8116	0,2466
P20029	Hspa5; Hspa8; Hspa2; Hspa1a; Hspa1l; Hspa1b	78 kDa glucose-regulated protein;Heat shock cognate 71 kDa protein;Heat shock-related 70 kDa protein 2;Heat shock 70 kDa protein 1A;Heat shock 70 kDa protein 1-like;Heat shock 70 kDa protein 1B	116056,7	187155,0	1,6126	0,2554
Q06318	Scgb1a1	Uteroglobin	239165000,0	269825000,0	1,1282	0,3335

Q80YC5	F12	Coagulation factor XII;Coagulation factor XIIa heavy chain;Coagulation factor XIIa light chain	1443210,0	1241867,5	0,8605	0,3627
P06684	C5	Complement C5;Complement C5 beta chain;Complement C5 alpha chain;C5a anaphylatoxin;Complement C5 alpha chain	794635,0	729257,5	0,9177	0,3991
P97361	Bpifa1	BPI fold-containing family A member 1	1820085,0	2033675,0	1,1174	0,4105
Q61114	Bpifb1	BPI fold-containing family B member 1	236383,3	229166,7	0,9695	0,4323
P35242	Sftpa1	Pulmonary surfactant-associated protein A	117649000,0	112480750,0	0,9561	0,4411
P50405	Sftpb	Pulmonary surfactant-associated protein B	30340500,0	31258750,0	1,0303	0,4526
Q5SQ27	Sec14l3	SEC14-like 3 (S, cerevisiae)	889800,0	876647,5	0,9852	0,4878
Q99JC1	Iglc3;Iglc2	Ig lambda-3 chain C region;Ig lambda-2 chain C region	2007675,0			
D3YY70	Fbxl12	F-box/LRR-repeat protein 12	4558900,0			
Q9ESB3	Hrg	Histidine-rich glycoprotein	253913,3			
P17182	Eno1;Eno3;Eno2	Alpha-enolase;Beta-enolase;Gamma-enolase;Enolase		117530,3		
E9Q9F7	Ccdc146	Coiled-coil domain-containing 146		587510,0		
A6H5Z3	Exoc6b	Exocyst complex component 6B		215860,0		
P24549	Aldh1a1	Retinal dehydrogenase 1		196170,0		
P17563	Selenbp1	Selenium-binding protein 1		543142,5		
P97821	Ctsc	Dipeptidyl peptidase 1;Dipeptidyl peptidase 1 exclusion domain chain;Dipeptidyl peptidase 1 heavy chain;Dipeptidyl peptidase 1 light chain	410456,7			
Q6URW6	Myh14	Myosin-14		13683,3		
O35099	Map3k5	Mitogen-activated protein kinase kinase kinase 5	386866,7			
P11499	Hsp90ab1	Heat shock protein HSP 90-beta		240463,5		
P56395	Cyb5a	Cytochrome b5		688900,0		
P33267	Cyp2f2	Cytochrome P450 2F2		188132,5		
Q61362	Chi3l1	Chitinase-3-like protein 1		281210,0		
A0JNT9	Ccdc64	Bicaudal D-related protein 1		4425480,0		
Q60692	Psmb6	Proteasome subunit beta type-6		373795,0		
P97298	Serpinf1	Pigment epithelium-derived factor		265670,0		
Q00623	Apoa1	Apolipoprotein A-I;Proapolipoprotein A-I;Truncated apolipoprotein A-I	228550,0			
O08749	Dld	Dihydrolipoyl dehydrogenase, mitochondrial	104739,7			
Q8BXK9	Clic5	Chloride intracellular channel protein 5	522456,7			
P33622	Apoc3	Apolipoprotein C-III		967073,3		

Q9Z1R9	Prss1	Protease, serine 1 (trypsin 1)	57690000,0

Table S2. Proteins found to associate with Fe_3O_4 NPs in Healthy and MetS conditions.

Shared Proteins ID	Unique Proteins in Fe3O4 NP-Healthy BC	Unique Proteins in Fe3O4 NP-MetS BC
P35242	D3YY70	A0JNT9
Q06318	O35099	Q9Z1R9
P60710	Q99JC1	E9Q9F7
P50405	Q8BXK9	P33267
P07724	P97821	P17563
P50404	Q00623	P11499
Q9DBR7	Q9ESB3	A6H5Z3
Q09XV5	O0874	P56395
P06728	-	P97298
P13020	-	Q60692
P05213	-	P33622
P68372	-	Q61362
Q00897	-	P24549
Q6GT24	-	P17182
P40936	-	Q6URW6
P09813	-	-
P14069	-	-
P06684	-	-
O09043	-	-
A8DUK4	-	-
A0A0R4J0I1	-	-
P08074	-	-
Q80YC5	-	-
O08553	-	-
O88456	-	-
P29699	-	-
P07309	-	-
Q5SQ27	-	-
Q00896	-	-
P01027	-	-
P10126	-	-
P16858	-	-
Q06890	-	-

P97361	-	-
Q60605	-	-
P68134	-	-
P52480	-	-
Q61114	-	-
P20029	-	-
Q8BHL4	-	-
P29788	-	-

Table S3. Comprehensive list of relative abundance differences for all lipids in BCs.

Lipid Class	Lipid ID	Precursor Ion -> Product Ion	Average Fe_3O_4 NP-Healthy BC	Average Fe_3O_4 NP-MetS BC	Fold Change Average Fe_3O_4 NP-Healthy BC	Fold Change Average Fe_3O_4 NP-MetS BC	p-value from Core Analysis
Diacylglycerol	DG 18:2_16:0	610,5 -> 313,5	15230	17031	1	1,1182	0,0138
Fatty Acid	C22:1	337,3 -> 337,3	38322	33320	1	0,8695	0,0138
Fatty Acid	C26:1	393,3 -> 393,3	67861	53269	1	0,7850	0,0185
Ceramides	Cer(d18:2/22:0)	620,6 -> 262,4	4168	3717	1	0,8918	0,0215
Phosphatidylcholines/Sphingomyelins	Lyso PC(15:0)	482,3168 -> 184,1	1838	1664	1	0,9050	0,0256
Diacylglycerol	DG 16:0_16:1	584,5 -> 311,5	21734	19182	1	0,8826	0,0293
Acyl Carnitines	(2E)-hexenedioylcarnitine, O-octanoylcarnitine	288,1 -> 85,1	2518	1891	1	0,7512	0,0310
Acyl Carnitines	(9Z)-3-hydroxydodecenoylcarnitine	358,3 -> 85,1	1623	1943	1	1,1975	0,0351
Phosphatidylglycerol	PG(32:1)	738,5015 -> 549,5015	18197	10096	1	0,5548	0,0375
Triglycerides	TG 20:0_34:0	908,9 -> 579,9	2751	2152	1	0,7823	0,0395
Phosphatidylglycerol	PG(32:2)	736,4859 -> 547,4859	2513	2270	1	0,9031	0,0430
Acyl Carnitines	2-Hydroxylauroylcarnitine, 3-hydroxydodecanoylcarnitine	360,3 -> 85,1	4131	3531	1	0,8548	0,0453
Phosphatidylglycerol	PG(30:0)	712,4859 -> 523,4859	4458	3397	1	0,7619	0,0473
Fatty Acid	C24:1	365,3 -> 365,3	42204	38552	1	0,9135	0,0519
Acyl Carnitines	(9Z)-3-hydroxydodecenoylcarnitine	358,3 -> 197,3	1027	868	1	0,8455	0,0530
Fatty Acid	C19:0	297,3 -> 297,3	198315	160945	1	0,8116	0,0533
Acyl Carnitines	(5Z,8Z)-tetradecadienoylcarnitine	368,3 -> 207,3	719	847	1	1,1779	0,0536
Acyl Carnitines	3-hydroxypalmitoleoylcarnitine, Heptadecanoyl carnitine	414,3 -> 355,3	981	773	1	0,7883	0,0547

Acyl Carnitines	O-(17-carboxyheptadecanoyl)carnitine	458,3 -> 85,1	1110	959	1	0,8637	0,0560
Triglycerides	TG 20:0_34:1	906,8 -> 577,8	3013	2066	1	0,6857	0,0601
Fatty Acid	C14:1	225,2 -> 225,2	88080	63867	1	0,7251	0,0619
Phosphatidylethanolamine	PE(32:1)	690,507 -> 549,507	4677	3493	1	0,7467	0,0635
Cholesterol Esters	20:0 Cholesteryl ester	698,6471 -> 369,1	1292	1232	1	0,9534	0,0644
Fatty Acid	C26:0	395,3 -> 395,3	56733	51048	1	0,8998	0,0660
Acyl Carnitines	, TetradecanoylcarnitineO-tetradecanoylcarnitine	372,3 -> 211,3	804	903	1	1,1230	0,0732
Triglycerides	TG 20:0_40:6	980,9 -> 651,9	2610	2090	1	0,8007	0,0762
Fatty Acid	C15:1	239,3 -> 239,3	128238	81576	1	0,6361	0,0791
Triglycerides	TG 18:0_36:3	902,8 -> 603,8	1835	2135	1	1,1637	0,0797
Phosphatidylcholines/Sphingomyelins	SM(d18:2/14:0)	673,5 -> 184,1	2076	1707	1	0,8224	0,0831
Phosphatidylethanolamine	PEo(36:5)	724,5277 -> 583,5277	2586	2361	1	0,9130	0,0899
Triglycerides	TG 18:0_34:3	874,8 -> 575,8	1705	1865	1	1,0943	0,0904
Phosphatidylcholines/Sphingomyelins	PC(30:2)	702,5 -> 184,1	2229	2004	1	0,8995	0,0904
Acyl Carnitines	(5Z,8Z)-tetradecadienoylcarnitine	368,3 -> 309,3	784	722	1	0,9213	0,0974
Phosphatidylcholines/Sphingomyelins	SM(d18:1/14:0)	675,5 -> 184,1	7843	3407	1	0,4344	0,0975
Acyl Carnitines	cis-5-Tetradecenoylcarnitine	370,3 -> 209,3	858	746	1	0,8699	0,0980
Ceramides	Cer(t18:0/24:0(2OH))	684,7 -> 282,4	13855	5396	1	0,3895	0,1017
Phosphatidylinositol	PI(34:5)	846,4863 -> 569,4863	6138	4833	1	0,7875	0,1056
Phosphatidylcholines/Sphingomyelins	PCo(38:5)	794,6 -> 184,1	1839	2027	1	1,1023	0,1144
Acyl Carnitines	(2E,4Z)-decadienoylcarnitine	312,2 -> 151,2	1028	910	1	0,8853	0,1168
Acyl Carnitines	Decanoylcarnitine	316,2 -> 155,2	1396	1683	1	1,2056	0,1250
Triglycerides	TG 18:1_34:3	874,8 -> 601,8	1651	1763	1	1,0680	0,1312
Ceramides	Cer(d14:1/20:0(2OH))	554,5 -> 208,3	4195	3845	1	0,9167	0,1312
Ceramides	Cer(d14:1/24:0(2OH))	610,6 -> 208,3	4644	3796	1	0,8173	0,1322
Acyl Carnitines	O-behenoylcarnitine	484,4 -> 323,4	883	809	1	0,9164	0,1330
Acyl Carnitines	O-malonylcarnitine, Hydroxybutyrylcarnitine	248,1 -> 85,1	1069	1232	1	1,1532	0,1336
Phosphatidylcholines/Sphingomyelins	Lyso PC(18:1)	522,3481 -> 184,1	2029	1899	1	0,9357	0,1354
Phosphatidylethanolamine	PEo(36:4)	726,5433 -> 585,5433	2560	2388	1	0,9328	0,1356
Acyl Carnitines	(5Z,8Z)-tetradecadienoylcarnitine	368,3 -> 60,1	787	724	1	0,9192	0,1358
Cholesterol Esters	22:1 Cholesteryl ester	724,6628 -> 369,1	1280	1184	1	0,9255	0,1417
Diacylglycerol	DG 16:1_18:1	610,5 -> 339,5	19396	15759	1	0,8125	0,1429
Phosphatidylserine	PSp(16:0)	482,2879 -> 297,2879	4197	3500	1	0,8338	0,1433
Ceramides	Cer(t18:0/20:0(2OH))	628,6 -> 282,4	7128	5119	1	0,7181	0,1447
Diacylglycerol	DG 16:1_16:1	582,5 -> 311,5	15593	13456	1	0,8630	0,1453

Ceramides	Cer(d18:0/20:0(2OH))	612,6 -> 266,4	13406	5897	1	0,4398	0,1461
Diacylglycerol	DG 16:0_18:2	610,5 -> 337,5	15550	16186	1	1,0409	0,1512
Diacylglycerol	DG 16:1_18:0	612,5 -> 341,5	20450	16023	1	0,7835	0,1571
Diacylglycerol	DG 16:0_18:1	612,5 -> 339,5	27540	24618	1	0,8939	0,1596
Phosphatidylcholines/Sphingomyelins	PC(36:8), PCo(36:1)	774,5 -> 184,1	1987	2191	1	1,1030	0,1598
Phosphatidylglycerol	PG(34:4)	760,4859 -> 571,4859	51012	38071	1	0,7463	0,1642
Acyl Carnitines	2-Hydroxylauroylcarnitine, 3-hydroxydodecanoylcarnitine	360,3 -> 199,3	866	788	1	0,9101	0,1672
Phosphatidylcholines/Sphingomyelins	Lyso PC(16:0)	496,3325 -> 184,1	3141	2734	1	0,8704	0,1697
Phosphatidylglycerol	PG(34:0)	768,5485 -> 579,5485	7276	5906	1	0,8118	0,1702
Phosphatidylcholines/Sphingomyelins	PCo(34:3)	742,6 -> 184,1	1938	1810	1	0,9339	0,1705
Triglycerides	TG 18:2_34:1	874,8 -> 577,8	1989	2143	1	1,0771	0,1757
Ceramides	Cer(d18:0/22:0)	624,6 -> 266,4	4851	4582	1	0,9446	0,1758
Triglycerides	TG 18:0_36:4	900,8 -> 601,8	1648	1739	1	1,0548	0,1764
Phosphatidylglycerol	PGo(34:0)	754,5692 -> 565,5692	2384	2273	1	0,9533	0,1782
Acyl Carnitines	(2E,5Z,7E)-decatrienoylcarnitine	310,2 -> 251,2	669	723	1	1,0815	0,1784
Acyl Carnitines	O-(11-carboxyundecanoyl)carnitine	374,2 -> 213,2	1112	1006	1	0,9042	0,1796
Triglycerides	TG 18:0_30:1	822,8 -> 523,8	1582	1762	1	1,1143	0,1820
Phosphatidylethanolamine	PE(38:6)	764,5226 -> 623,5226	4488	4927	1	1,0977	0,1825
Phosphatidylcholines/Sphingomyelins	SM(d18:0/14:0)	677,6 -> 184,1	2286	1930	1	0,8443	0,1854
Triglycerides	TG 18:1_34:2	876,8 -> 603,8	1763	2000	1	1,1344	0,1893
Cholesterol Esters	18:0 Cholesteryl ester	670,6158 -> 369,1	1566	1477	1	0,9430	0,1949
Phosphatidylglycerol	PG(34:1)	766,5328 -> 577,5328	62595	47633	1	0,7610	0,1957
Diacylglycerol	DG 16:0_18:0	614,5 -> 341,5	109461	103456	1	0,9451	0,1965
Triglycerides	TG 18:1_32:3	848,8 -> 575,8	1722	1892	1	1,0988	0,1970
Acyl Carnitines	(4Z)-decenoylcarnitine, 9-Decenoylcarnitine, CIS-4-DECENOYL CARNITINE	314,2 -> 85,1	1401	1277	1	0,9114	0,2013
Diacylglycerol	DG 18:0_16:0	614,5 -> 313,5	115684	108822	1	0,9407	0,2021
Phosphatidylcholines/Sphingomyelins	PC(30:1)	704,5 -> 184,1	10324	8239	1	0,7980	0,2031
Phosphatidylcholines/Sphingomyelins	PCo(32:2)	716,6 -> 184,1	2879	2559	1	0,8889	0,2082
Phosphatidylglycerol	PG(34:5)	758,4702 -> 569,4702	9356	7657	1	0,8184	0,2105
Phosphatidylethanolamine	PE(34:4)	712,4913 -> 571,4913	61381	47912	1	0,7806	0,2116
Acyl Carnitines	3-hydroxyarachidonoylcarnitine	464,3 -> 303,3	680	648	1	0,9530	0,2193
Ceramides	Cer(d18:1/16:0)	538,5 -> 264,3	4136	3958	1	0,9571	0,2197
Triglycerides	TG 16:0_34:4	846,8 -> 575,8	1609	1823	1	1,1335	0,2201
Acyl Carnitines	Decanoylcarnitine	316,2 -> 257,2	1000	1165	1	1,1651	0,2203

Acyl Carnitines	(2E)-hexenedioylcarnitine, O-octanoylcarnitine	288,1 -> 127,1	1020	944	1	0,9255	0,2247
Triglycerides	TG 16:0_32:1	822,8 -> 551,8	1786	2109	1	1,1810	0,2256
Acyl Carnitines	2-Hydroxymyristoylcarnitine, 3-hydroxytetradecanoylcarnitine	388,3 -> 227,3	841	779	1	0,9267	0,2265
Phosphatidylethanolamine	PE(34:3)	714,507 -> 573,507	3960	3539	1	0,8936	0,2276
Phosphatidylinositol	PI(34:4)	848,5019 -> 571,5019	25052	20112	1	0,8028	0,2277
Phosphatidylcholines/Sphingomyelins	PC(30:0)	706,5 -> 184,1	160534	122542	1	0,7633	0,2304
Phosphatidylcholines/Sphingomyelins	SM(d18:1/18:1)9Z))	729,6 -> 184,1	2441	2671	1	1,0943	0,2309
Acyl Carnitines	3-hydroxylinoleoylcarnitine,	440,3 -> 381,3	846	784	1	0,9268	0,2342
Diacylglycerol	DG 16:1_16:0	584,5 -> 313,5	20043	18833	1	0,9396	0,2344
Phosphatidylcholines/Sphingomyelins	Lyso PC(3:0)	314,129 -> 184,1	1725	1679	1	0,9732	0,2346
Triglycerides	TG 18:1_32:2	850,8 -> 577,8	2229	2619	1	1,1747	0,2367
Phosphatidylglycerol	PG(32:0)	740,5172 -> 551,5172	27774	22452	1	0,8084	0,2406
Phosphatidylserine	PS(34:3)	758,4968 -> 573,4968	2724	2576	1	0,9459	0,2445
Acyl Carnitines	O-(17-carboxyheptadecanoyl)carnitine	458,3 -> 144,1	692	721	1	1,0425	0,2466
Acyl Carnitines	2-ethylacryloylcarnitine, Tiglylcarnitine	244,1 -> 83,1	25502	32922	1	1,2910	0,2476
Phosphatidylcholines/Sphingomyelins	PC(36:0), PCp(38:6)	790,6 -> 184,1	2578	2755	1	1,0688	0,2536
Triglycerides	TG 18:0_34:2	876,8 -> 577,8	1956	2164	1	1,1063	0,2549
Phosphatidylcholines/Sphingomyelins	PCo(38:6)	792,6 -> 184,1	2378	2695	1	1,1333	0,2550
Acyl Carnitines	, TetradecanoylcarnitineO-tetradecanoylcarnitine	372,3 -> 313,3	717	755	1	1,0522	0,2565
Acyl Carnitines	Stearidonyl carnitine	420,3 -> 144,1	700	667	1	0,9526	0,2615
Acyl Carnitines	(2E,4Z)-decadienoylcarnitine	312,2 -> 85,1	1013	1108	1	1,0939	0,2637
Phosphatidylethanolamine	PE(34:2)	716,5226 -> 575,5226	5775	5128	1	0,8879	0,2638
Phosphatidylcholines/Sphingomyelins	SM(d16:1/24:1)	785,6 -> 184,1	4645	4075	1	0,8773	0,2654
Phosphatidylethanolamine	PE(34:5)	710,4757 -> 569,4757	10993	9374	1	0,8527	0,2665
Phosphatidylcholines/Sphingomyelins	PC(38:6)	806,6 -> 184,1	23709	30002	1	1,2654	0,2720
Phosphatidylethanolamine	PE(34:1)	718,5383 -> 577,5383	7360	7962	1	1,0817	0,2726
Phosphatidylcholines/Sphingomyelins	PC(38:7), PCo(38:0)	804,6 -> 184,1	2063	2208	1	1,0701	0,2740
Phosphatidylcholines/Sphingomyelins	Lyso PC(6:0)	356,176 -> 184,1	2751	2505	1	0,9104	0,2759
Acyl Carnitines	12-Hydroxy-12-octadecanoylcarnitine, 3-hydroxyoctadecanoylcarnitine	444,4 -> 283,4	777	739	1	0,9503	0,2763
Phosphatidylcholines/Sphingomyelins	PCo(34:4)	740,6 -> 184,1	20624	17231	1	0,8355	0,2819

Acyl Carnitines	O-palmitoleoylcarnitine, trans-Hexadec-2-enoyl carnitine	398,3 -> 339,3	1281	1463	1	1,1417	0,2847
Acyl Carnitines	2-ethylacryloylcarnitine, Tiglylcarnitine	244,1 -> 85,1	2320	2678	1	1,1542	0,2853
Phosphatidylcholines/Sphingomyelins	SM(d18:1/24:0)	815,7 -> 184,1	2541	2402	1	0,9453	0,2901
Phosphatidylcholines/Sphingomyelins	PC(34:2)	758,6 -> 184,1	103035	128550	1	1,2476	0,2902
Phosphatidylcholines/Sphingomyelins	PCo(34:0)	748,6 -> 184,1	6446	7603	1	1,1795	0,2927
Phosphatidylserine	PS(34:4)	756,4811 -> 571,4811	21482	18308	1	0,8522	0,2927
Phosphatidylglycerol	PGo(32:0)	726,5379 -> 537,5379	2290	2360	1	1,0309	0,2935
Acyl Carnitines	(7Z,10Z)-hexadecadienoylcarnitine	396,3 -> 85,1	1079	1148	1	1,0638	0,2959
Acyl Carnitines	Valerylcarnitine, Isovalerylcarnitine	246,2 -> 187,2	843	811	1	0,9626	0,2966
Acyl Carnitines	cis-5-Tetradecenoylcarnitine	370,3 -> 311,3	704	731	1	1,0393	0,2978
Phosphatidylcholines/Sphingomyelins	Lyso PC(16:1)	494,3168 -> 184,1	1820	1760	1	0,9668	0,2987
Phosphatidylcholines/Sphingomyelins	SM(d18:0/16:0)	705,6 -> 184,1	5901	5175	1	0,8771	0,2997
Phosphatidylcholines/Sphingomyelins	SM(d18:1/20:0)	759,6 -> 184,1	41549	50941	1	1,2260	0,2997
Phosphatidylcholines/Sphingomyelins	Lyso PC(18:2)	520,3325 -> 184,1	1770	1711	1	0,9669	0,3036
Acyl Carnitines	(2E,5Z,7E)-decatrienoylcarnitine	310,2 -> 85,1	1507	1648	1	1,0931	0,3060
Acyl Carnitines	cis-5-Tetradecenoylcarnitine	370,3 -> 85,1	1344	1425	1	1,0605	0,3078
Triglycerides	TG 16:0_32:3	818,8 -> 547,8	1627	1709	1	1,0507	0,3115
Phosphatidylcholines/Sphingomyelins	PC(36:7), PCo(36:0)	776,5 -> 184,1	1803	1862	1	1,0328	0,3134
Triglycerides	TG 18:0_32:2	850,8 -> 551,8	1847	1979	1	1,0713	0,3178
Phosphatidylcholines/Sphingomyelins	PC(32:5)	724,5 -> 184,1	2252	2138	1	0,9495	0,3188
Phosphatidylglycerol	PG(36:5)	786,5015 -> 597,5015	2407	2331	1	0,9683	0,3209
Phosphatidylinositol	PI(34:3)	850,5176 -> 573,5176	2690	2586	1	0,9616	0,3211
Acyl Carnitines	Propenoylcarnitine	216,1 -> 55,1	1299	1388	1	1,0687	0,3222
Phosphatidylcholines/Sphingomyelins	SM(d18:2/22:1)	783,6 -> 184,1	16783	19832	1	1,1817	0,3251
Phosphatidylinositol	PI(38:6)	900,5332 -> 623,5332	2668	2596	1	0,9730	0,3279
Phosphatidylethanolamine	PE(36:4)	740,5226 -> 599,5226	6001	6653	1	1,1087	0,3286
Phosphatidylcholines/Sphingomyelins	PC(34:5)	752,5 -> 184,1	12513	10893	1	0,8706	0,3296
Phosphatidylcholines/Sphingomyelins	PC(36:4)	782,6 -> 184,1	37698	44701	1	1,1858	0,3298
Phosphatidylcholines/Sphingomyelins	PCo(36:2)	772,6 -> 184,1	2010	2103	1	1,0466	0,3305
Phosphatidylglycerol	PG(36:4)	788,5172 -> 599,5172	10576	9413	1	0,8901	0,3325
Phosphatidylglycerol	PG(38:6)	812,5172 -> 623,5172	7131	6453	1	0,9050	0,3331
Acyl Carnitines	Butenylcarnitine	230,1 -> 69,1	1120	1184	1	1,0569	0,3333
Phosphatidylcholines/Sphingomyelins	PCo(36:5)	766,6 -> 184,1	2200	2110	1	0,9592	0,3337
Triglycerides	TG 16:0_34:3	848,8 -> 577,8	1850	1962	1	1,0607	0,3370

Phosphatidylcholines/Sphingomyelins	PC(40:7), PCo(40:0)	832,6 -> 184,1	2038	1952	1	0,9575	0,3412
Phosphatidylcholines/Sphingomyelins	PC(38:4)	810,6 -> 184,1	7948	9075	1	1,1418	0,3418
Phosphatidylcholines/Sphingomyelins	SM(d18:0/18:0)	733,6 -> 184,1	259582	224869	1	0,8663	0,3435
Phosphatidylcholines/Sphingomyelins	PC(34:4)	754,5 -> 184,1	70943	62017	1	0,8742	0,3475
Acyl Carnitines	(5Z,8Z)-tetradecadienoylcarnitine	368,3 -> 85,1	1335	1398	1	1,0473	0,3481
Phosphatidylserine	PS(34:5)	754,4655 -> 569,4655	5372	4978	1	0,9267	0,3497
Phosphatidylcholines/Sphingomyelins	PC(32:1)	732,6 -> 184,1	676960	590445	1	0,8722	0,3504
Phosphatidylcholines/Sphingomyelins	PC(38:5)	808,6 -> 184,1	9363	10596	1	1,1317	0,3529
Phosphatidylcholines/Sphingomyelins	Lyso PC(10:0)	412,2386 -> 184,1	1739	1785	1	1,0264	0,3534
Triglycerides	TG 16:0_32:2	820,8 -> 549,8	1938	2131	1	1,0992	0,3569
Phosphatidylcholines/Sphingomyelins	PCo(32:1)	718,6 -> 184,1	11742	10487	1	0,8931	0,3600
Phosphatidylcholines/Sphingomyelins	SM(d16:1/22:1)	757,6 -> 184,1	5109	5636	1	1,1032	0,3603
Phosphatidylcholines/Sphingomyelins	PC(36:5)	780,6 -> 184,1	3572	3824	1	1,0706	0,3630
Phosphatidylcholines/Sphingomyelins	PC(36:3)	784,6 -> 184,1	10709	9698	1	0,9056	0,3690
Diacylglycerol	DG 18:0_18:0	642,6 -> 341,6	432619	419881	1	0,9706	0,3703
Phosphatidylcholines/Sphingomyelins	SM(d18:2/20:1)	755,6 -> 184,1	16700	15061	1	0,9019	0,3705
Phosphatidylglycerol	PG(36:2)	792,5485 -> 603,5485	4401	4132	1	0,9387	0,3709
Acyl Carnitines	(11Z,14Z)-eicosadienoylcarnitine	452,4 -> 85,1	1342	1399	1	1,0424	0,3779
Phosphatidylcholines/Sphingomyelins	PC(40:6)	834,6 -> 184,1	4176	4511	1	1,0801	0,3798
Acyl Carnitines	(9Z)-3-hydroxyoctadecenoylcarnitine	442,3 -> 85,1	1036	996	1	0,9612	0,3820
Phosphatidylcholines/Sphingomyelins	SM(d18:2/24:1)	811,7 -> 184,1	5086	5491	1	1,0796	0,3824
Acyl Carnitines	(5Z,8Z)-3-hydroxytetradecadienoylcarnitine	384,3 -> 223,3	745	760	1	1,0207	0,3828
Acyl Carnitines	(2E,5Z,7E)-decatrienoylcarnitine	310,2 -> 149,2	1795	1693	1	0,9433	0,3881
Phosphatidylcholines/Sphingomyelins	PC(36:2)	786,6 -> 184,1	7331	6847	1	0,9340	0,3889
Triglycerides	TG 18:0_32:4	846,8 -> 547,8	1624	1659	1	1,0219	0,3906
Phosphatidylcholines/Sphingomyelins	PC(40:4)	838,6 -> 184,1	1855	1887	1	1,0176	0,3909
Triglycerides	TG 18:1_34:1	878,8 -> 605,8	1776	1740	1	0,9798	0,3932
Acyl Carnitines	(9Z,12Z,15Z)-3-hydroxyoctadecatrienoylcarnitine	438,3 -> 277,3	704	692	1	0,9826	0,3950
Phosphatidylcholines/Sphingomyelins	PC(38:3)	812,6 -> 184,1	2934	3069	1	1,0460	0,3951
Acyl Carnitines	O-(13-carboxytridecanoyl)carnitine	402,3 -> 241,3	728	741	1	1,0170	0,3991
Phosphatidylcholines/Sphingomyelins	PCp(36:5)	764,6 -> 184,1	3310	3171	1	0,9580	0,3995
Triglycerides	TG 18:1_30:1	822,8 -> 549,8	2057	2179	1	1,0593	0,4103
Phosphatidylglycerol	PG(38:5)	814,5328 -> 625,5328	4850	4631	1	0,9550	0,4105

Acyl Carnitines	O-oleoylcarnitine, Elaidic carnitine	426,4 -> 85,1	1234	1258	1	1,0191	0,4108
Phosphatidylserine	PSo(16:0)	484,3035 -> 299,3035	2720	2676	1	0,9838	0,4110
Phosphatidylcholines/Sphingomyelins	PCo(32:0)	720,6 -> 184,1	18734	17416	1	0,9296	0,4111
Phosphatidylglycerol	PG(38:4)	816,5485 -> 627,5485	3528	3414	1	0,9677	0,4130
Phosphatidylcholines/Sphingomyelins	PCo(34:2)	744,6 -> 184,1	3344	3205	1	0,9586	0,4139
Diacylglycerol	DG 16:0_16:0	586,5 -> 313,5	44194	45139	1	1,0214	0,4142
Phosphatidylcholines/Sphingomyelins	PC(32:2)	730,5 -> 184,1	16722	15622	1	0,9342	0,4154
Phosphatidylinositol	PI(36:4)	876,5332 -> 599,5332	2785	2729	1	0,9798	0,4168
Phosphatidylcholines/Sphingomyelins	SM(d18:1/16:0)	703,6 -> 184,1	6181	5907	1	0,9557	0,4196
Phosphatidylcholines/Sphingomyelins	PC(34:3)	756,6 -> 184,1	10644	10089	1	0,9479	0,4282
Phosphatidylcholines/Sphingomyelins	PCo(36:4)	768,6 -> 184,1	2286	2242	1	0,9807	0,4323
Phosphatidylcholines/Sphingomyelins	SM(d18:0/20:0)	761,6 -> 184,1	80718	76141	1	0,9433	0,4325
Phosphatidylcholines/Sphingomyelins	PC(32:3)	728,5 -> 184,1	3227	3134	1	0,9714	0,4333
Phosphatidylcholines/Sphingomyelins	SM(d16:1/24:0)	787,7 -> 184,1	4159	4272	1	1,0272	0,4334
Phosphatidylcholines/Sphingomyelins	PC(32:0), PCp(32:4)	734,6 -> 184,1	1009132	1072711	1	1,0630	0,4354
Acyl Carnitines	Stearidonyl carnitine	420,3 -> 85,1	1701	1685	1	0,9906	0,4372
Phosphatidylcholines/Sphingomyelins	PC(40:5)	836,6 -> 184,1	2363	2328	1	0,9853	0,4465
Phosphatidylcholines/Sphingomyelins	PCo(38:4)	796,6 -> 184,1	1943	1965	1	1,0113	0,4498
Phosphatidylinositol	PIo(20:5)	632,3869 -> 355,3869	2726	2701	1	0,9908	0,4520
Phosphatidylcholines/Sphingomyelins	SM(d16:1/18:1)	701,6 -> 184,1	2715	2673	1	0,9849	0,4539
Phosphatidylglycerol	PG(36:1)	794,5641 -> 605,5641	3248	3204	1	0,9865	0,4546
Phosphatidylcholines/Sphingomyelins	PC(34:6)	750,5 -> 184,1	2163	2139	1	0,9890	0,4555
Ceramides	Cer(d18:0/24:0)	652,7 -> 266,4	6111	5988	1	0,9798	0,4567
Phosphatidylglycerol	PG(34:2)	764,5172 -> 575,5172	31632	30531	1	0,9652	0,4569
Acyl Carnitines	(2E)-octenoylcarnitine, 2-octenoyl-L-carnitine	286,2 -> 144,1	685	678	1	0,9901	0,4581
Phosphatidylglycerol	PG(34:3)	762,5015 -> 573,5015	4479	4397	1	0,9815	0,4624
Acyl Carnitines	Propionylcarnitine	218,1 -> 57,1	876	870	1	0,9930	0,4637
Phosphatidylcholines/Sphingomyelins	Lyso PC(19:3)	532,3325 -> 184,1	2291	2268	1	0,9900	0,4644
Diacylglycerol	DG 18:1_16:0	612,5 -> 313,5	21215	21364	1	1,0070	0,4645
Phosphatidylcholines/Sphingomyelins	Lyso PC(18:0)	524,3638 -> 184,1	2799	2759	1	0,9857	0,4646
Acyl Carnitines	3-hydroxypalmitoleoylcarnitine, Heptadecanoyl carnitine	414,3 -> 253,3	770	764	1	0,9914	0,4655
Phosphatidylcholines/Sphingomyelins	PC(34:1)	760,6 -> 184,1	202811	208374	1	1,0274	0,4702
Phosphatidylglycerol	PG(36:3)	790,5328 -> 601,5328	4043	4093	1	1,0123	0,4716
Phosphatidylcholines/Sphingomyelins	PCo(34:1)	746,6 -> 184,1	9788	9611	1	0,9820	0,4754

Acyl Carnitines	(4Z)-decenoylcarnitine, 9-Decenoylcarnitine, CIS-4-DECENOYL CARNITINE	314,2 -> 255,2	862	869	1	1,0082	0,4791
Acyl Carnitines	2-ethylacryloylcarnitine, Tiglylcarnitine	244,1 -> 60,1	894	898	1	1,0054	0,4815
Phosphatidylcholines/Sphingomyelins	PC(34:0)	762,6 -> 184,1	30854	31254	1	1,0130	0,4842
Acyl Carnitines	Palmitoylcarnitine, (5Z)-13-carboxytridec-5-enoylcarnitine	400,3 -> 239,3	756	759	1	1,0049	0,4846
Triglycerides	TG 16:1_36:0	878,8 -> 577,8	1670	1675	1	1,0031	0,4856
Triglycerides	TG 18:1_30:0	824,8 -> 551,8	2363	2359	1	0,9982	0,4933
Acyl Carnitines	Acetyl-carnitine	204,1 -> 43,1	1829	1833	1	1,0019	0,4941
Phosphatidylcholines/Sphingomyelins	SM(d18:1/18:0)	731,6 -> 184,1	11772	11795	1	1,0020	0,4959

Figure S1. (a-h) 1h Total protein for (a) STAT3 & p-STAT3 (b) p38 & p-p38 (c) NF-κB & p-NF-κB (d) ERK & p-ERK. 24h Total protein for (e) STAT3 & p-STAT3 (f) p38 & p-p38 (g) ERK & p-ERK (h) NF-κB & p-NF-κB.

1h Total protein for:

a) STAT3 & p-STAT3

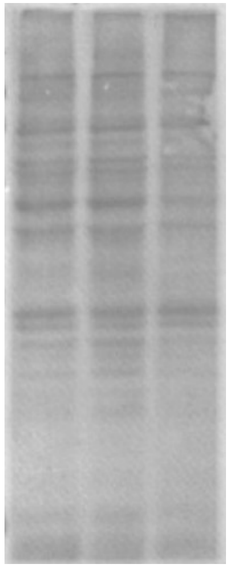

b) p38 & p-p38

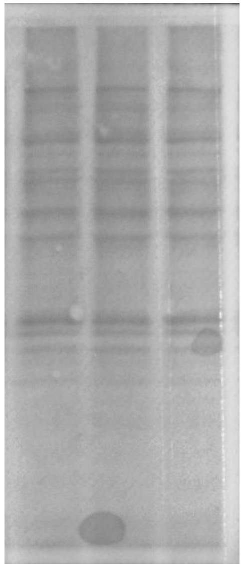

c) NF-κB & p-NF-κB

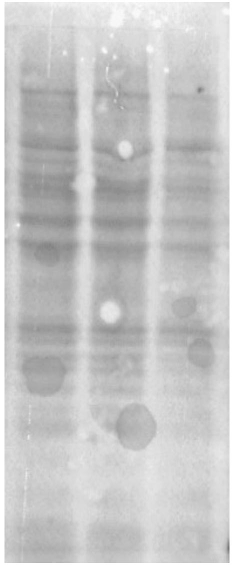

d) ERK & p-ERK

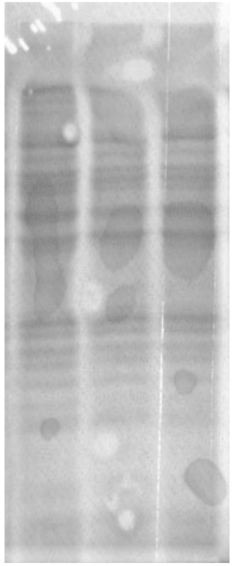

24h Total protein for:

e)STAT3 & p-STAT3

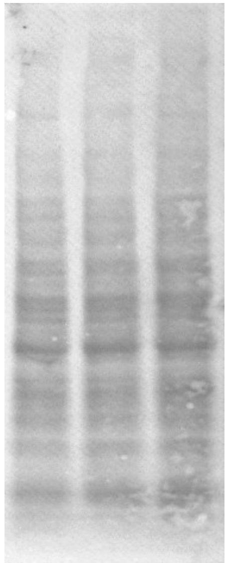

f)p38 & p-p38

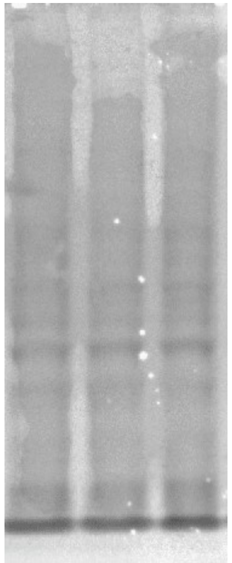

g)ERK & p-ERK

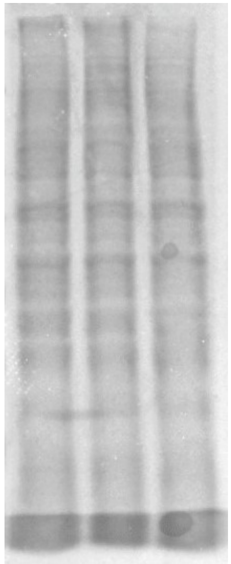

h)NF-κB & p-NF-κB

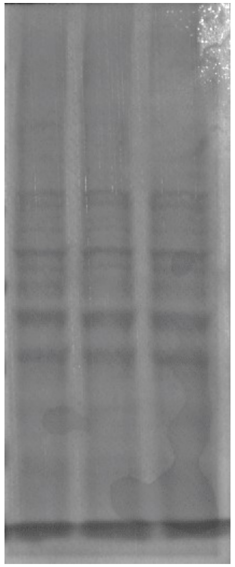

Supplement: Supplementary file 1 [file nanomaterials-12-02022-s001.zip › nanomaterials-1733347-supplementary.pdf]
